# Generation of Antigenic Diversity in *Plasmodium falciparum* by Structured Rearrangement of *Var* Genes During Mitosis

**DOI:** 10.1371/journal.pgen.1004812

**Published:** 2014-12-18

**Authors:** Antoine Claessens, William L. Hamilton, Mihir Kekre, Thomas D. Otto, Adnan Faizullabhoy, Julian C. Rayner, Dominic Kwiatkowski

**Affiliations:** 1 Malaria Programme, Wellcome Trust Sanger Institute, Hinxton, United Kingdom; 2 MRC Centre for Genomics and Global Health, University of Oxford, Oxford, United Kingdom; Weill Medical College of Cornell University, United States of America

## Abstract

The most polymorphic gene family in *P. falciparum* is the ∼60 *var* genes distributed across parasite chromosomes, both in the subtelomeres and in internal regions. They encode hypervariable surface proteins known as *P. falciparum* erythrocyte membrane protein 1 (PfEMP1) that are critical for pathogenesis and immune evasion in *Plasmodium falciparum*. How *var* gene sequence diversity is generated is not currently completely understood. To address this, we constructed large clone trees and performed whole genome sequence analysis to study the generation of novel *var* gene sequences in asexually replicating parasites. While single nucleotide polymorphisms (SNPs) were scattered across the genome, structural variants (deletions, duplications, translocations) were focused in and around *var* genes, with considerable variation in frequency between strains. Analysis of more than 100 recombination events involving *var* exon 1 revealed that the average nucleotide sequence identity of two recombining exons was only 63% (range: 52.7–72.4%) yet the crossovers were error-free and occurred in such a way that the resulting sequence was in frame and domain architecture was preserved. *Var* exon 1, which encodes the immunologically exposed part of the protein, recombined in up to 0.2% of infected erythrocytes *in vitro* per life cycle. The high rate of *var* exon 1 recombination indicates that millions of new antigenic structures could potentially be generated each day in a single infected individual. We propose a model whereby *var* gene sequence polymorphism is mainly generated during the asexual part of the life cycle.

## Author Summary

Malaria kills >600,000 people each year, with most deaths caused by *Plasmodium falciparum*. A family of proteins known as *P. falciparum* erythrocyte membrane protein 1, PfEMP1, is expressed on the surface of infected erythrocytes and plays an important role in pathogenesis. Each *P. falciparum* genome contains approximately 60 highly polymorphic *var* genes encoding the PfEMP1 proteins, and monoallelic expression with periodic switching results in immune evasion. *Var* gene polymorphism is thus critical to this survival strategy. We investigated how *var* gene diversity is generated by performing an *in vitro* evolution experiment, tracking *var* gene mutation in ‘real-time’ with whole genome sequencing. We found that genome structural variation is focused in and around *var* genes. These genetic rearrangements created new ‘chimeric’ *var* gene sequences during the mitotic part of the life cycle, and were consistent with processes of mitotic non-allelic homologous recombination. The recombinant *var* genes were always in frame and with conserved overall *var* gene architecture, and the recombination rate implies that many millions of rearranged *var* gene sequences are produced every 48-hour life cycle within infected individuals. In conclusion, we provide a detailed description of how new *var* gene sequences are continuously generated in the parasite genome, helping to explain long-term parasite survival within infected human hosts.

## Introduction

*Plasmodium falciparum* is a unicellular parasite that causes malaria in humans. It infects over 300 million people per year and is estimated to have killed 600,000–1.2 million people in 2010 [1]. One of the most remarkable biological features of *P. falciparum* is an exceptionally polymorphic parasite antigen expressed on the surface of infected erythrocytes, known as *P. falciparum* erythrocyte membrane protein 1 (PfEMP1) [2]. PfEMP1 is encoded by a family of hypervariable genes known as *var*, each representing a different antigenic form, and the parasite is able to vary its antigenic profile by switching expression between different *var* genes [3]. This allows the parasite to evade the human immune system and has major clinical consequences, as PfEMP1 mediates the cellular interactions and pathological properties of infected erythrocyte [4]–[6].

**Figure 1:**
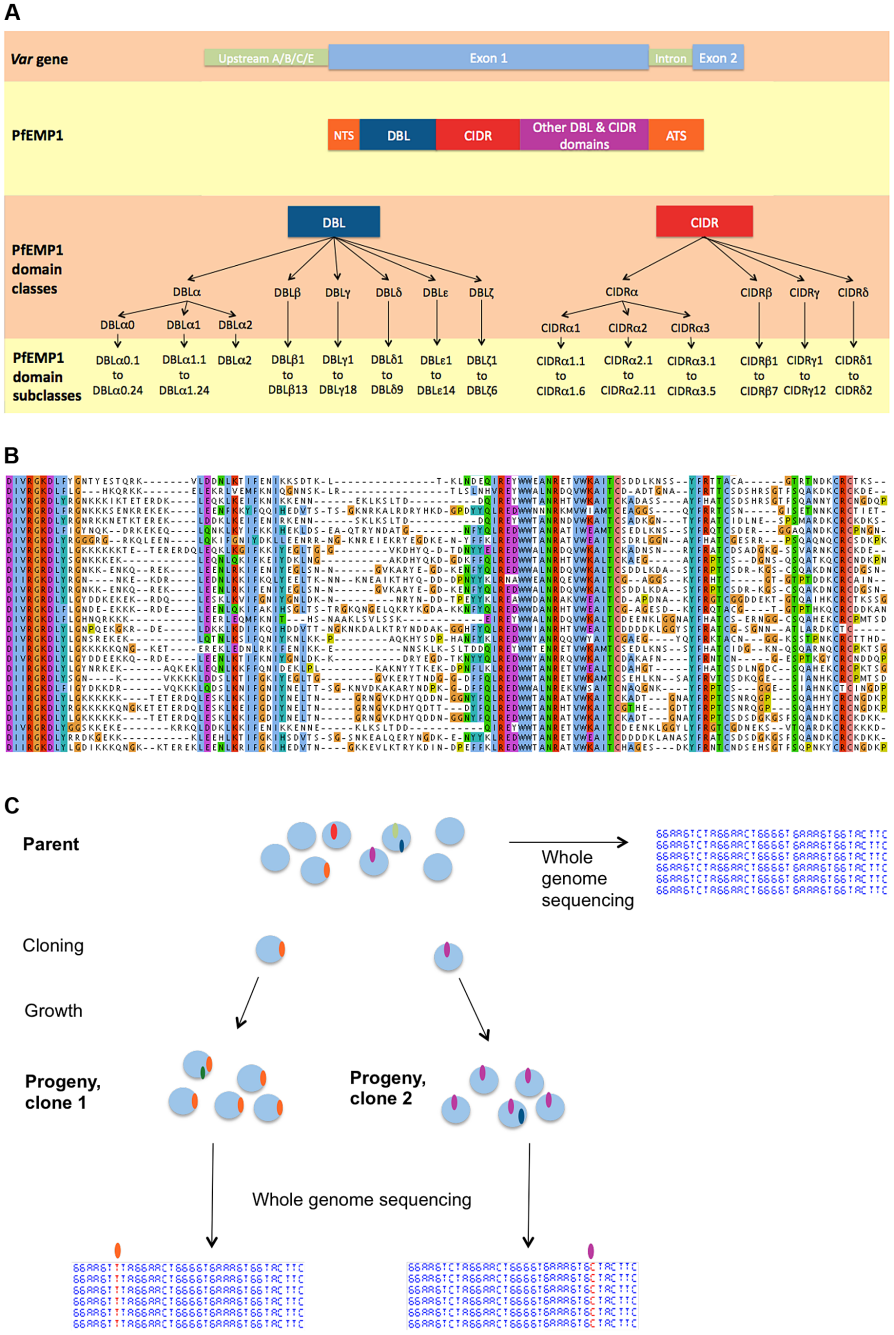
*Var* gene nomenclature and generation of a clone tree. (A) *Var* gene nomenclature, as defined in [8]. From top to bottom: schematic *var* gene locus, schematic PfEMP1 protein with its DBL & CIDR classes followed by subclasses. Note that the minor domain classes CIDRα4 to α6 and the *var2csa* specific domains are not represented here. Abbreviations: DBL =  Duffy-binding like; CIDR =  Cys rich inter-domain region; NTS = N-terminal segment; ATS =  Acidic terminal segment. (B) Partial alignment of 3D7 PfEMP1 sequences, representative of the juxtaposition of conserved and variable stretches of amino acids. (C) Principle of generating a clone tree: each blue circle represents a parasite genome, a coloured oval is a mutation, Single Nucleotide Polymorphism (SNP) or Structural variant. Cloning by limiting dilution randomly selects one individual parasite, possibly with one or more mutations. Note that mutations observed in a progeny sample occurred within the parental generation, at some point after the preceding clonal dilution.

Each parasite genome contains approximately 60 *var* genes distributed in clusters across most of the 14 chromosomes. Based on conserved sequences upstream of the coding region, *var* genes are divided into three main groups. Group A *var* genes, which are confined to subtelomeric regions, have been shown by *in vivo* gene expression studies to be involved in the pathogenesis of severe malaria [2]. Group C *var* genes are found only in internal chromosomal regions while group B *var* genes occur both within chromosomes and at the subtelomere. Despite being the most polymorphic gene family in *P. falciparum*, *var* genes share broad structural similarities and some conserved motifs (Fig. 1A). The first exon (4–10 kb) begins with an N-terminal segment (NTS) and is followed by a succession of Duffy Binding Like (DBL) and cysteine rich interdomain regions (CIDR) domains. The second exon is semi-conserved and encodes the intracellular component of PfEMP1. Based on distance tree analysis, DBL domains are subdivided into six major classes (DBLα, β, γ, δ, ε, ζ) and CIDR domains into four (CIDRα, β, γ, δ) [7]. Each class can then be further subdivided into subclasses (DBLα0.1, DBLα0.2, etc) [8].

Previous studies have observed ectopic (non-allelic) recombination between subtelomeric *var* genes [9]–[13] and it has been proposed that this might account for a large part of the *var* gene diversity observed within and between species [14]. However it is not known whether *var* gene recombination is sufficiently frequent to be the primary driver of *var* gene diversity, nor whether this recombination occurs mainly in meiosis, which takes place in the mosquito, or during mitosis, which includes the entirety of the intraerythrocytic stages within the human host. It is also unclear whether recombination obeys particular patterns, and how this might relate to *var* gene structure. Understanding how the parasite manages to generate such an extreme level of sequence diversity while preserving the overall architecture and biological functionality of the *var* gene repertoire is fundamental to understanding *P. falciparum* pathogenesis.

We used an experimental evolution approach to systematically investigate the mechanisms that drive *var* gene diversity, performing whole genome sequence analysis on>200 clonal parasites. Parasites cultured *in vitro* in human erythrocytes were regularly sub-cloned to isolate single infected red blood cells, so that mutations arising in asexually dividing cells could be detected using whole genome sequencing of the expanded progeny derived from these cells (Fig. 1C). We generated these ‘clone trees’ for geographically diverse *P. falciparum* strains, comparing parasite genomes separated by 20–30 cycles of replication (S1 Fig.). The scale of our dataset allowed us to perform a comprehensive analysis of mitotic *var* gene recombination using observed recombination events for the first time. This revealed how *var* gene sequence polymorphism – and, by inference, parasite antigenic variation – can be continuously generated during asexual division over the course of a single human infection.

## Results

### Structural variation is focused in and around *var* genes in 3D7

**Figure 2:**
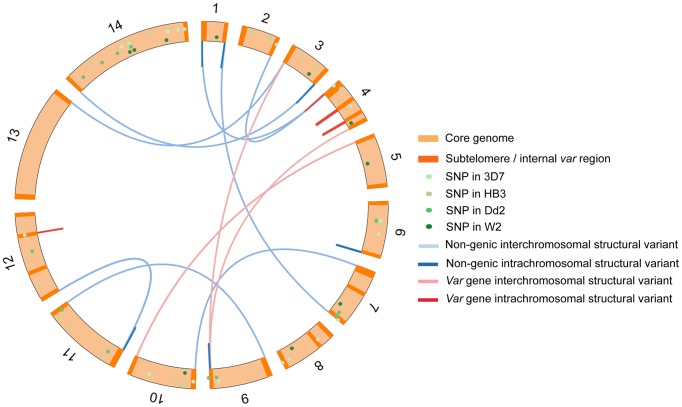
Chromosomal location of structural variants in the 3D7 clone tree and SNPs in the 3D7, Dd2, W2 and HB3 clone trees. SNPs from 3D7, Dd2, W2 and HB3 clone tress were scattered around the genome. In contrast, structural variants (deletions, duplications and translocations) were solely in subtelomeric and internal *var* gene regions. Structural variants in Dd2, W2 and HB3 *var* genes are not shown here as their *var* gene location in these strains is unknown.

We began by analysing the 3D7 strain of *P. falciparum* as this has the most complete genome assembly of any lab isolate. 19 individual parasites were cloned from 3D7 by limiting dilution and cultured until sufficient parasite quantity was reached for whole genome sequencing. From 2 of these 19 subclones, another 3 rounds of clonal dilutions were performed over a combined culturing period of 203 days (S1 Fig.). Whole-genome sequencing of a total of 37 subclones identified 20 *de novo* single nucleotide substitutions (hereafter referred to as Single Nucleotide Polymorphisms or SNPs) distributed throughout the genome, and 40 *de novo* structural variations comprising 10 duplications, 8 deletions and 22 translocations (Fig. 2, S2–S4 Table). Our analysis focused solely on *de novo* mutations, i.e. found in one or more subclone(s) but not in the parental clone. As expected, the first generation of subclones contained more mutations than subsequent generations, because they were derived from a standard 3D7 culture that had been growing for several months prior to beginning our experiment. Of the 19 structural variations that affected coding regions, all were in *var* genes. Of the remaining 21 structural variations, all were in telomeric regions or within internal regions of chromosomes that contained clusters of *var* genes. From these data we concluded that *de novo* structural variations of the *P. falciparum* genome occur relatively frequently during the mitotic intraerythrocytic life cycle and are highly concentrated in and around *var* genes.

**Figure 3:**
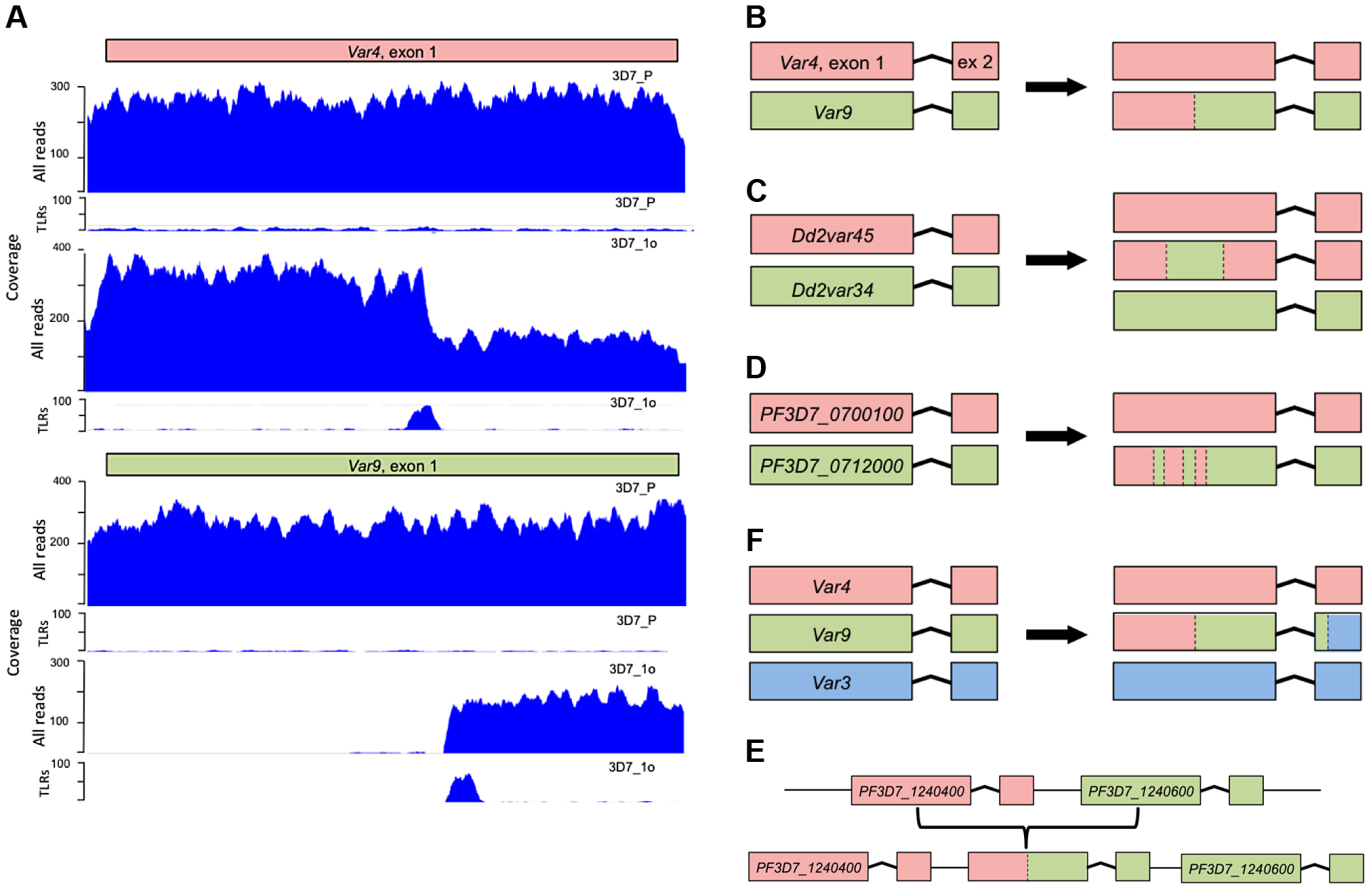
Examples of chimeric *var* genes created by recombination. (A) A typical *var* gene exon 1 translocation in 3D7. Clone 3D7_1o has zero coverage in the first half of its “*var9*” (PF3D7_0937800), with a peak of trans-locus reads (TLRs) at the cut-off point (the ‘parent’, 3D7_P is shown for comparison). The TLRs from *var9* are mate-paired with a TLR peak in exon 1 of “*var4*” (PF3D7_0426000), indicating that, in 3D7_1o, the first half of *var4* is fused with the second half of *var9*. There is ∼2x increase in coverage upstream of the TLR peak in *var4*. This suggests a gene conversion producing a new chimeric var4/9 sequence, with the first half of *var4* and the second half of var9 joined together. This is summarized with a cartoon in (B). (C) Example of a double recombination between two adjacent internal *var* genes (*Dd2var34* and *Dd2var45*) leading to a new chimeric *var* gene named *var34-45-34* (see main text and Fig. 4). (D) As depicted by this cartoon, the majority of recombining pairs of *var* genes showed multiple crossover points (S4 Table). (E) Triple reassortment *var* gene, composed of three ‘parent’ *var* genes (S2 Fig.). (F) ‘Duplication chimera’ in internal *var* genes. Duplicated sections of internal *var* genes recombine to form new chimeras. TLR =  Trans-locus reads. PCR and capillary sequencing data validating these chimeric sequences can be found in S2–S7 Fig.

Detailed inspection of these structural variations revealed that they took many forms (Fig. 3B–F) but all involved ectopic recombination between different *var* genes located either in the immediate vicinity on the same chromosome, or on different chromosomes. For example, Fig. 3A&B and S2 Fig. show a gene conversion event involving two subtelomeric *var* genes, in which the end subtelomere of chromosome 4 was duplicated and replaced the subtelomere of chromosome 9. The translocation breakpoints occurred within *var* genes on these two chromosomes, referred to as *var4* and *var9*, creating a chimeric *var4/var9* sequence. This gene conversion event was confirmed by capillary sequencing of PCR products, and the *var4*/*var9* chimera was subsequently inherited by downstream parasite clones. There were also instances of two *var* genes recombining with multiple crossover points (Fig. 3D, S3 & S4 Fig.) and recombination between more than two *var* genes (Fig. 3E and Fig. S2). Recombination in subtelomeric *var* genes involved both reciprocal exchange and non-reciprocal gene conversion in which the entire end of one chromosome was duplicated and replaced the corresponding section of the other chromosome. In addition to recombination between subtelomeric *var* genes on different chromosomes, we found recombination between internal *var* genes on the same chromosome (Fig. 3F, S5 & S6 Fig.).

### *Var* gene exon 1 recombination is a frequent mitotic event in diverse *P. falciparum* strains

To establish whether mitotic *var* gene recombination is a common feature among other *P. falciparum* strains, we generated similar clone trees for the Dd2 and W2 strains of drug-resistant *P. falciparum* from Southeast Asia, and for the HB3 strain of drug-sensitive *P. falciparum* from Honduras (S1 Fig.). The Dd2, W2 and HB3 clone trees were generated with a total of 7, 3, and 8 cloning rounds respectively, over a total of 298, 119 and 250 days. This generated 56, 20 and 81 subclones respectively, all of which were whole genome sequenced. Altogether, we observed 20, 11 and 19 SNPs in the Dd2, W2 and HB3 clone trees respectively, scattered across the genome (Fig. 2). As any mutation identified in a clone would have actually occurred in the previous generation, we can only use clones from the second generation onwards for calculating mutation rates (Fig. 1C). From these data we estimate mutation rates of 4.07×10^−10^, 3.63×10^−10^ and 3.78×10^−10^ SNPs per erythrocytic life cycle per nucleotide for 3D7, Dd2 and HB3 respectively (Table 1). The W2 clone tree did not contain enough clones for a reliable mutation rate estimate. Despite geographic and drug-sensibility differences between 3D7, Dd2 and HB3, there is no statistically significant difference in SNP mutation rate between these strains (Kruskal-Wallis, p>0.7); See Methods & Table 1 for rate calculations. The rates calculated here are similar to estimates for 3D7 and Dd2 by Bopp *et al* 2013, suggesting that SNP mutation rates are relatively constant across *P. falciparum* strains.

**Table 1:** Calculation of the Single Nucleotide Polymorphism mutation rate.

Column	A	B	C	D	E	F	G	H	I	J	K	L	M	N
	Generation	Number of clones	Number of SNPs	SNP/clone	Culturing (days)	Culturing (hours)	Assumed lifecycle time (hours)	Number of lifecycles ( = F/G)	SNP/lifecycle ( = D/H)	Genome size (Mb)	SNP/life cycle/bp ( = I/J)	Weighting*	Weighted SNP/lifecycle/bp ( = K/L)	Weighted SNP/lifecycle ( = I/L)
**3D7**	1	19	15	0.794	unkn.									
	2m	5	0	0	74	1776	48	37	0	23.3	0	0.278	0	0
	2o	7	2	0.29	79	1896	48	39.5	0.0072	23.3	3.10E–10	0.389	1.21E–10	2.81E–03
	3	6	3	0.5	50	1200	48	25	0.02	23.3	8.58E–10	0.333	2.86E–10	6.67E–03
	**Total**	**37**	**20**		**203**								**4.069E–10**	**9.480E–03**
**Dd2**	1	5	7	1.40	unkn.									
	(A)_2	10	5	0.50	64	1536	44.1	34.83	0.0144	23.3	6.16E–10	0.196	1.21E–10	2.81E–03
	(B)_2	17	4	0.24	64	1536	44.1	34.83	0.0068	23.3	2.90E–10	0.333	9.66E–11	2.25E–03
	(A)_3	3	1	0.33	47	1128	44.1	25.58	0.0130	23.3	5.59E–10	0.059	3.29E–11	7.67E–04
	(B)_3	2	0	0	47	1128	44.1	25.58	0	23.3	0	0.039	0	0
	(A)_4	14	3	0.21	41	984	44.1	22.31	0.0096	23.3	4.12E–10	0.275	1.13E–10	2.64E–03
	(B)_4	5	0	0	35	840	44.1	19.05	0	23.3	0	0.098	0	0
	**Total**	**56**	**20**		**298**								**3.635E–10**	**8.470E–03**
**HB3** (Two clone trees)	1	7	0	0	unkn.									
	2	8	0	0	29	696	49.7	14.00	0	23.3	0	0.119	0	0
	3	22	5	0.23	32	768	49.7	15.45	0.0147	23.3	6.31E–10	0.328	2.07E–10	4.83E–03
	4	15	1	0.067	50	1200	49.7	24.14	0.0028	23.3	1.19E–10	0.224	2.65E–11	6.18E–04
	5	8	2	0.25	47	1128	49.7	22.70	0.0110	23.3	4.73E–10	0.119	5.64E–11	1.32E–03
	b1	7	8	1.143	unkn.									
	b2	8	1	0.125	48	1152	49.7	23.18	0.0054	23.3	2.31E–10	0.119	2.76E–11	6.44E–04
	b3	6	2	0.33	44	1056	49.7	21.25	0.0157	23.3	6.73E–10	0.090	6.03E–11	1.40E–03
	**Total**	**81**	**19**		**250**								**3.782E–10**	**8.812E–03**

Each column is labelled to indicate the steps leading to the mutation rate numbers (see Methods for more details). We first calculate the number of SNPs per sub-clone detected in each clone tree generation. This represents the average number of *de novo* mutations in each clonal population (e.g. if the SNP/clone is 0.2, then approximately 1 in 5 clones will have a SNP). We divide this by the number of intraerythrocytic life cycles the parasites have undergone prior to the most recent cloning step, counting from the previous clonal dilution. The number of intraerythrocytic life cycles is reached by dividing the time in culture by the time per intraerythrocytic life cycle. This gives the SNPs/intraerythrocytic parasite life cycle for each clone tree generation. To derive the SNPs/life cycle/nucleotide, we divide by the haploid nuclear genome of *P. falciparum* (23,300,000 base pairs). The final mutation rate for each strain is an average from all the generations in the clone tree. We weight this average based on the number of subclones analysed in each generation, so that generations with more subclones have proportionally more representation in the average. Note that the first generation of subclones cannot be used to calculate the SNP mutation rate because the length of time in culture prior to our starting the experiment is not known. The W2 clone tree was too small to accurately determine the SNP mutation rate. The same process was used to calculate the exon 1 *var* recombination rates (S5 Table).

A complete high-quality genome assembly is currently only available for 3D7, which makes mapping difficult in other strains, especially in *var* genes. For these strains we therefore focused only on a detailed analysis of recombination events in *var* exon 1, which encodes the extracellular domains responsible for antigenic diversity. In Dd2 and W2, we observed 11 and 13 instances of *var* exon 1 recombinations in 7 and 6 pairs of recombining *var* genes respectively (Table 2). In contrast, there were no recombination events in HB3 clones, despite similar breadth and length of the clone trees and despite the *var* gene assemblies of HB3 and Dd2 being of a comparable standard.

**Table 2:** Summary of *var* gene exon 1 recombinations.

	Clones analysed*	Total number of SNPs	Pairs of *var* genes involved (of which are internal *vars*)	Total number of recombinations in *var* exon 1	var/SNP ratio**
**3D7 clone tree**	31	20	5 (4)	17	0.27
**Dd2 clone tree**	56	20	7 (5)	11	0.35
**W2 clone tree**	20	11	6 (6)	13	0.54
**HB3 clone tree**	81	23	0	0	0
**3D7xHB3**	16	6	6 (3)	14	1
**HB3xDd2**	30	12	6 (2)	24	0.50
**7G8xGB4**	27	12	16 (unknown)	28	1.33
**Bopp ** ***et al*** [9] **	15	/	2 (0)	2	/
**Guler ** ***et al*** [15]	5	/	1 (1)	1	/
**Total**	**281**		**46**	**110**	

* Excluding the parental samples. ** var/SNP ratio is the number of pairs of recombining *var* genes divided by the number of SNPs.

**Although Bopp *et al* reports 4 pairs of recombining *var* genes, one is in the exon2, a region that was not considered in this analysis; another one did not correlate with our own analysis and was discarded. All but one chimeric *var* genes reported in Sander *et al* were also discovered in our analysis (the remaining one was is in a progeny sample not available to us).

We estimate that the rate at which pairs of *var* genes undergo exon 1 recombination in the Dd2 clone tree is 2.32×10^−3^ per erythrocytic life cycle, compared with 9.48×10^−3^, 8.47×10^−3^ and 8.81×10^−3^ SNPs per life cycle in 3D7, Dd2 and HB3 respectively (see Methods for rate calculations). This means that in the Dd2 strain, for every 48-hour intraerythrocytic generation approximately 0.2% of parasites will have undergone a *var* exon 1 recombination event producing a new chimeric *var* gene.

We further analysed the *var* gene recombination rate by comparing the ratio of total *de novo var* recombinations to SNPs observed in the clone trees. Our mutation rate calculations given above exclude mutations found in the first generation of each clone tree because these mutations occurred prior to our beginning the experiment after an unknown length of time in culture (Fig. 1C). However, mutations identified in this first clone tree round must have arisen during the intraerythrocytic stage, at some point between the original cloning of 3D7/HB3/Dd2 in the 1980s and the start of our clone tree experiments. As demonstrated in this study and in Bopp *et al* [9], the SNP mutation rate appears to be relatively constant between strains. Therefore, the number of SNPs found in first generation subclones becomes a proxy for the time of culture of the parent, i.e. the more SNPs identified, the longer the parent had been in culture since the original cloning step. Because of this constant SNP mutation rate, we can use all subclones, including the first generations subclones, to measure the ‘*var* recombination/SNP ratio’, which indicates the number of *var* recombinations over a period of time. The overall *var* exon 1 recombination to SNP mutation ratio is 0.25, 0.35, 0.54 and 0 in the 3D7, Dd2, W2 and HB3 clone trees respectively (Table 2). Thus, while the SNP mutation rate is constant between different *P. falciparum* strains, the structural variant rate in HB3 *var* genes is significantly lower than in Dd2 (p = 0.03, Fisher's exact test), at least with the specific HB3 isolate used in our laboratory.

**Figure 4:**
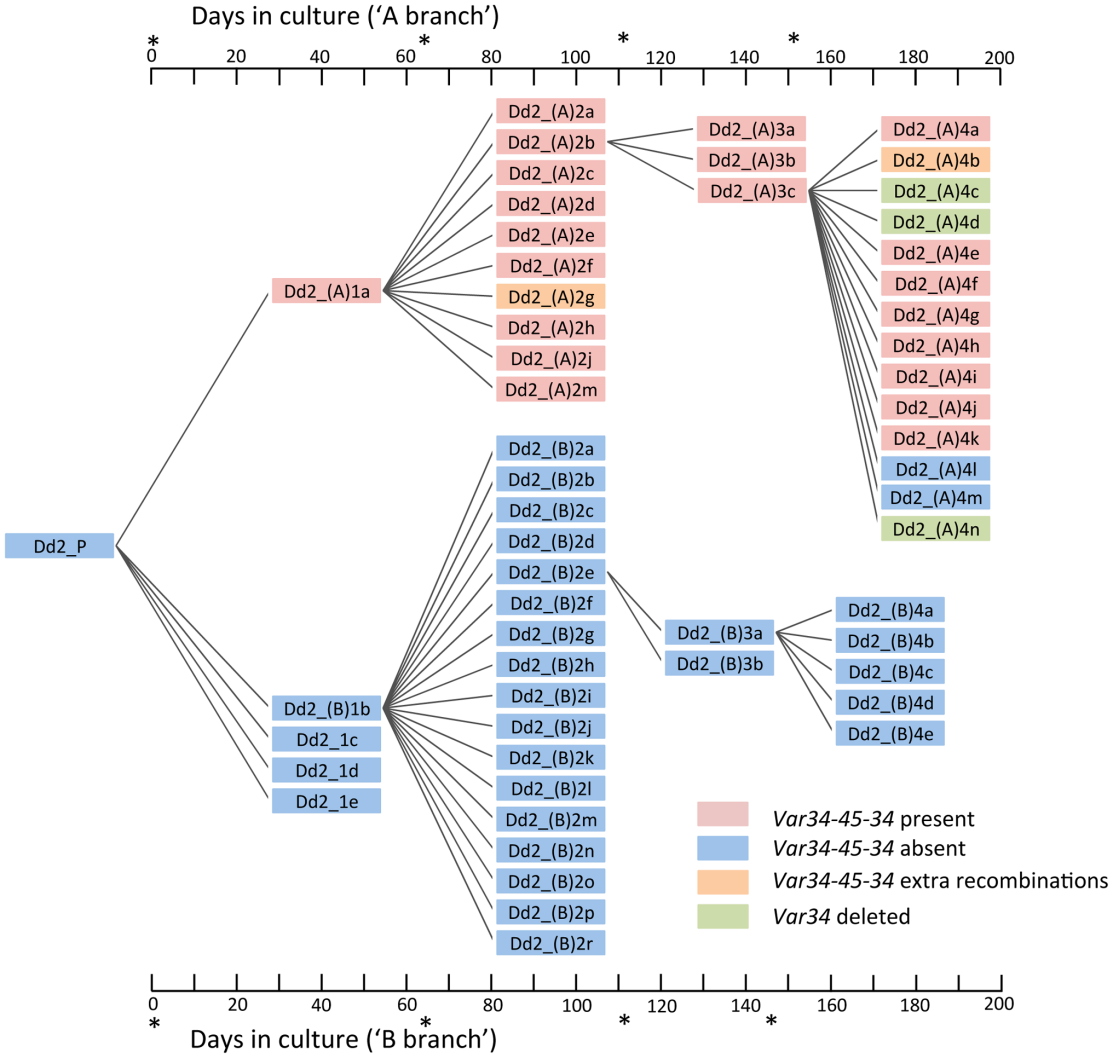
The Dd2 clone tree. (see S1 Fig. for 3D7/HB3/W2 clone trees). Cloning by limiting dilution randomly selects one individual parasite, possibly with one or more mutations. Note that mutations observed in a progeny sample occurred within the parental generation, at some point after the preceding clonal dilution. In this Dd2 clone tree, clones Dd2_(A)1a (A-branch) and Dd2_(B)1b (B-branch) were arbitrarily selected for further clonal dilutions. As described in the main text, a double recombination event between *Dd2var34* and *Dd2var45* genes created a new chimeric *var* named *var34/45/34* in clone Dd2_(A)1a. All subsequent subclones framed in red also showed *var34/45/34*, while two subclones (Dd2_(A)4l & 4m) had lost it, two subclones in orange showed extra recombinations involving *var34/45/34* and three subclones in green had lost the original *Dd2var34* copy. *denotes limiting dilution date.

The dynamic nature of *var* recombination in Dd2 is illustrated by a pair of internal *var* genes referred to here as *var34* and *var45* (Fig. 4). Recombination between *var34* and *var45* was first observed in a first generation subclone of the Dd2 clone tree (Dd2_(A)1a). This was a double crossover recombination which created a new chimeric *var* gene (‘*var34/var45*’) while retaining the original versions of *var34* and var*45* (Fig. 3C, Fig. 4, S7 Fig.). Over the next three generations of the clone tree, most of the 31 descendants of the recombined clone inherited *var34*, *var45* and the *var34/var45* chimera, with a few exceptions. Three descendants lost the *var34* sequence, two lost the *var34/var45* chimera, and two underwent further recombination of *var34* and *var45* producing new chimeras. In contrast, we observed no changes involving *var34* or *var45* in the branch of the clone tree that was not descended from the clone in which recombination between *var34* and *var45* had first occurred.

### *Var* gene exon 1 recombination is structured such that overall *var* gene architecture is preserved

The clone trees demonstrate that *var* gene recombination during intraerythrocytic growth is extensive and complex, and in at least three strains occurs nearly as frequently as SNPs. To investigate the patterns and mechanisms of *var* gene recombination, we collated all available data on chromosomal crossovers in *var* exon 1 (Table 1) based on our clone tree experiments, together with data from previous smaller studies [9], [15] as well as data obtained by sequencing the progeny of three genetic crosses (Dd2xHB3, 3D7xHB3 and 7G8xGB4) [16]–[18] (Pearson & Miles, in prep) (discussed in more detail below). From these combined datasets we identified 110 crossovers in 46 pairs of *var* gene exon 1s, generating a mean of 2.4 (range 1–12) crossover points per recombining pair of exons (Table 2, S4 and S8 Fig.).

**Figure 5:**
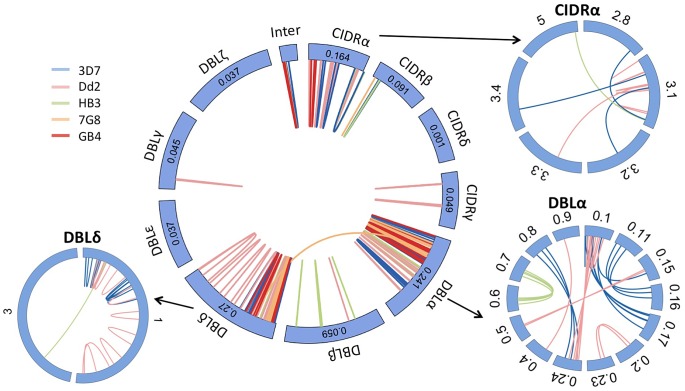
*Var* gene recombination occurs within domain classes and between domain subclasses. Each *var* gene typically comprises 4 to 7 DBL and CIDR domains, each domain being subdivided in various classes (α, β,…) and subclasses (α0.1, α0.2,…). In all but one of the recombining *var* pairs, the crossover breakpoint was predicted between domains of the same class in both genes. In contrast, recombination was indiscriminate of subclass within each domain class (outside wheels). Subclassification data are not available for 7G8 and GB4 strains. For clarity only recombining domain subclasses are shown here. Inter  =  Interdomain region. Numbers in the central wheel indicate the relative frequency of that domain class in group B and C *var* genes. DBLα domains showed the highest number of recombinations (S13 Fig.) but within that domain we found no evidence for a hotspot (S14 Fig.).

These data revealed a highly structured pattern of ectopic recombination. We found that all of the crossovers were in frame and in the vast majority of cases (109/110) the recombining sequences were of the same domain class, e.g. CIDRα almost invariably recombined with CIDRα (Fig. 5). Every *var* gene begins with a DBLα and CIDRα domain, followed by a variable number and order of other domains. Given that recombination only occurs between the same domain classes, this mechanism would preserve the overall architecture of chimeric *var* genes. Consistent with this, all observed chimeric *var* genes possessed a typical number of *var* domains. We observed only one exception to this, between *7G8var49* and *7G8var56*, in which recombination took place in an area of shared homology between a DBLα and a DBLδ domain [8], producing a truncated gene with only two domains (S8 Fig.).

**Figure 6:**
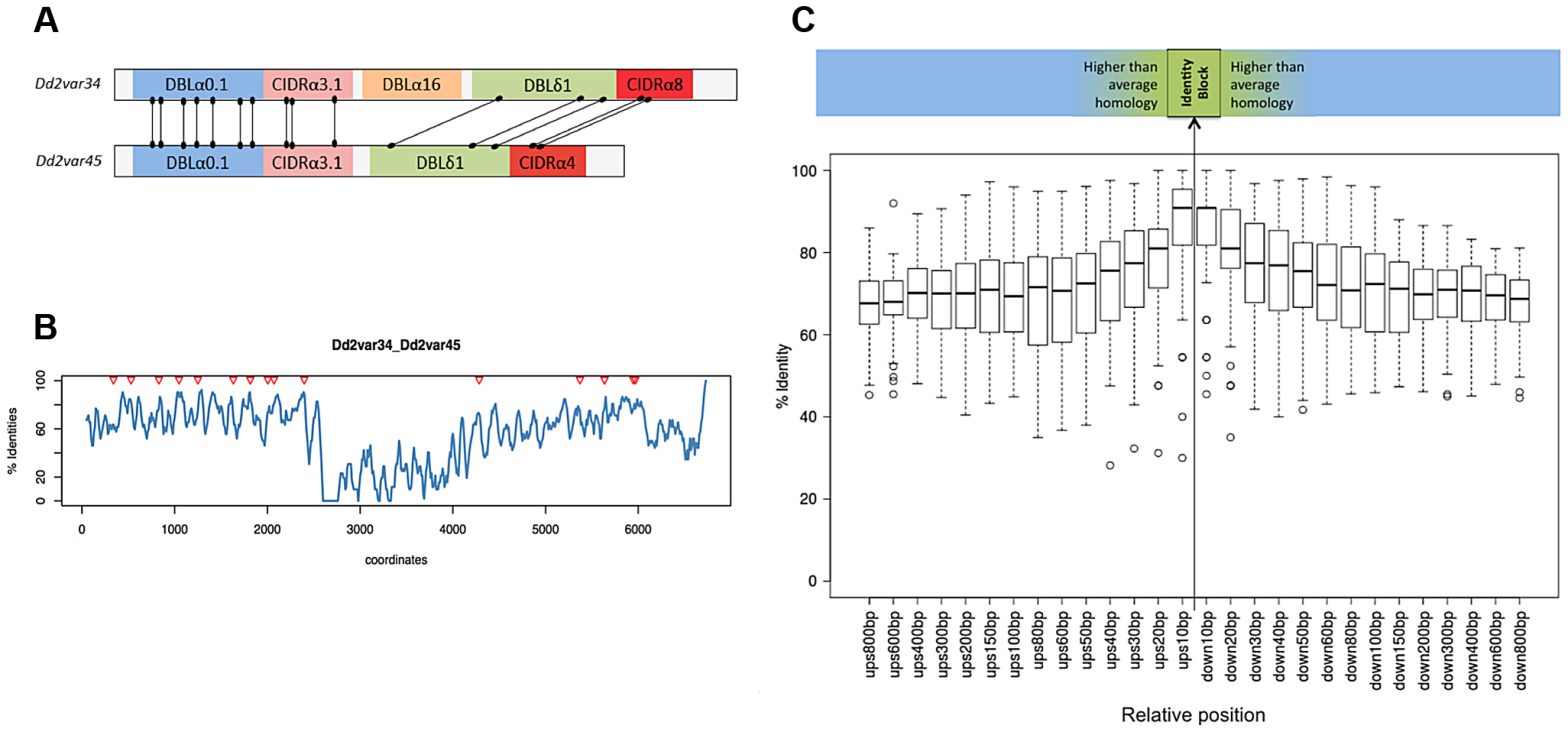
Recombination occurs in shared *var* domains within identity blocks. (A) and (B) refer to two example *var* genes, *Dd2var34* and *Dd2var45*. For all *var* gene pairs, see S8 and S12 Fig. (A) All recombination crossovers observed between the two *var* genes shown schematically, from the Dd2 clone tree and HB3xDd2 cross. (B) Sequence homology using overlapping 50 bp windows following a ClustalW alignment of *var* exon 1. Triangles indicate coordinates of crossover midpoints within the alignment. (C) Median percentage identity for all recombinations between sequences of 10–800 bp upstream (‘ups’) and downstream (‘down’) from the recombination breakpoint, defined as the identity block midpoint. Homology peaks at the point of recombination.

The new gene sequences generated by this structured process were extremely diverse, as *var* genes did not necessarily recombine with the most highly homologous *var* genes to themselves (S9 Fig.). The average sequence identity of the two recombining domains was 68%, and that of the two recombining exons was only 63%. In the terminology used by Rask et al, 65% of recombinations were between domains of different sub-classes (Fig. 6). But despite this overall diversity, we observed that crossovers invariably took place within a short section of perfect sequence identity ranging in length from 4 to 48 bp (median 15 bp). This section of sequence identity, which we refer to here as an identity block (S10 Fig.), was typically located within a broader section of elevated sequence homology (median 80 bp) relative to the average for the two genes concerned (Fig. 6). When exon 1 *var* sequences were aligned by BLAST analysis, 94% of crossovers were found to occur in a region of high similarity between the two recombining genes (S11 Fig.), whereas only 59% would be expected if the point of recombination was random (*P*<10^−5^ by chi-squared test).

We compared the recombination rates found in group A, B and C *var* genes from our pooled *var* exon 1 recombination dataset. In total, we observed 19 instances of B×B recombination, 12 instances of C×C recombination and 2 instances of B×C recombination. In contrast we observed no instances of recombination involving group A *var* genes. The 3D7 reference genome contains 10 group A, 37 group B and 13 group C *var* genes, so these observations depart significantly from what would be expected if the pairing of ectopic sequences was random across all *var* genes, (p = <0.0028, Fisher's exact test). These data suggest that group A *var* genes have a lower recombination rate than group B or C.

### Analysis of experimental genetic crosses indicates that *var* gene recombination occurs mainly during mitosis

**Figure 7:**
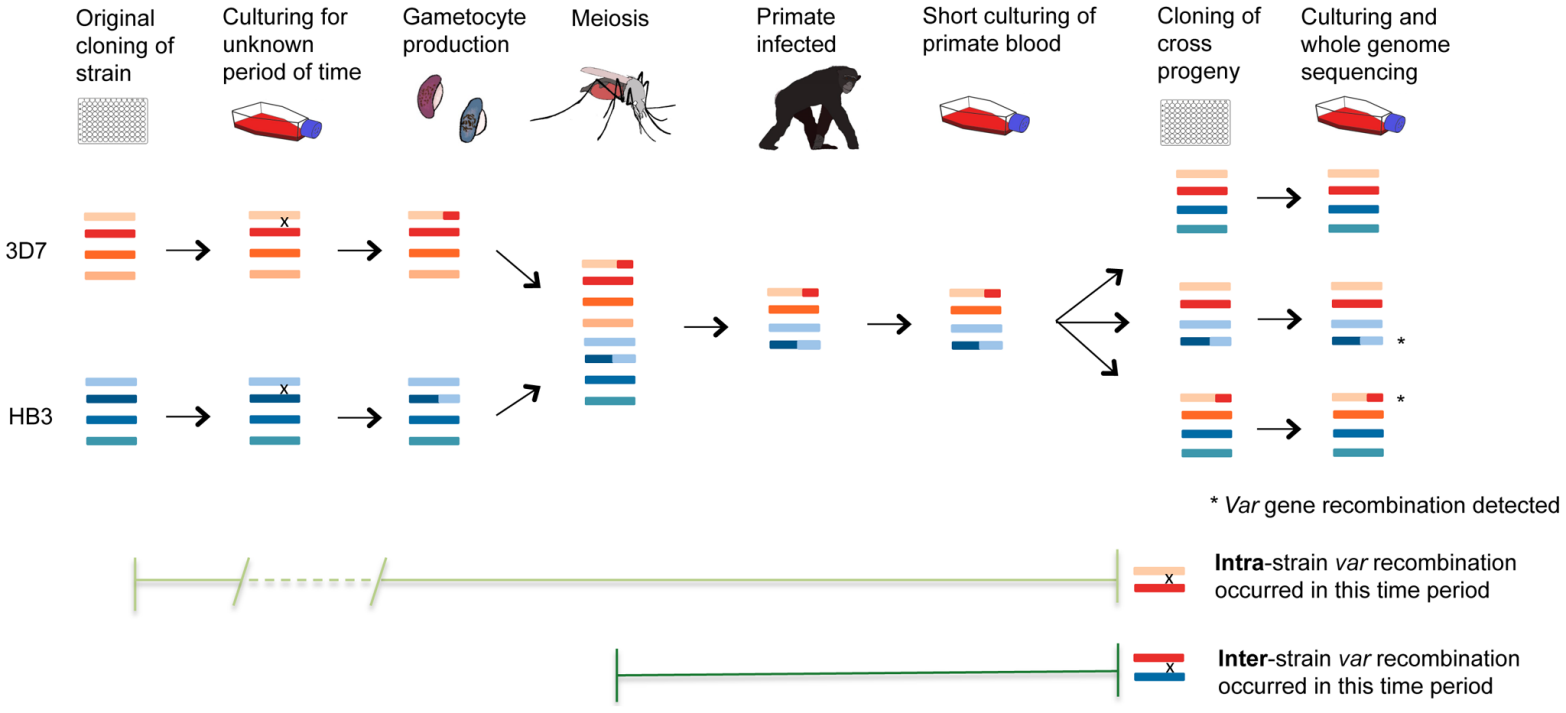
*Var* recombination in crosses progeny. *P. falciparum* experimental crosses use parasites that have been grown in culture, dividing asexually, for a variable length of time following their initial isolation from malaria patients. Mitotic *var* gene recombination could take place throughout this period, as seen in our clone trees. In the cross, parasite sexual forms are taken up by mosquitoes where gamete fusion occurs and the parasite becomes briefly diploid. Meiotic division ensues before the parasite becomes haploid again and divides mitotically for the remainder of its life cycle. The parasites are passed from the mosquito through splenectomized chimpanzees for progeny collection followed by sub-cloning and whole genome sequencing of expanded progeny (Miles *et al*, in prep.). Any mutations observed in the crosses progeny could thus have arisen either in the many rounds of mitosis before and after the cross, or in the single round of meiosis in the mosquito. Comparison with our clone tree data is strongly supportive of the former explanation for *var* gene recombination (see text). The fact that 26/28 of observed recombining *var* gene pairs in the progeny are of the same parental origin (‘intra-strain’) suggests that most of the mutations occurred during mitosis when the parents were being cultured prior to their uptake by the mosquito.

The prevailing view of *var* recombination is that it occurs primarily during meiosis, based on the observation of recombination in the progeny of experimental crosses [10], [19]. An alternative model is that *var* recombination in these progeny actually occurred during mitosis while the parasites were cultured *in vitro* prior to and after the cross itself [20]. To test these alternatives we identified and characterised *var* recombinations in genome sequence data from progeny of all three experimental genetic crosses, and compared them with analogous data from progeny of our clone trees. This comparison strongly supported the latter model – that the vast majority of *var* recombination occurs during mitosis – for three main reasons (Fig. 7). First, the recombination in both the (purely mitotic) clone trees and the crosses progeny share key features and appear to have been produced through the same process. Recombined *var* genes were always in frame without SNPs or InDels; crossovers took place in small regions of sequence identity; overall *var* exon 1 domain architecture was preserved because domains only recombined with other domains of the same class (with one exception), and recombination often involved multiple crossovers between the same two *var* genes, with an average of 2.6 and 2.4 crossovers per recombining *var* pair in the clone trees and crosses progeny respectively (S7 Table). Second, within the crosses progeny we could find only two cases where the *var* genes in a recombining *var* pair were derived from different parents. This is not what would be expected if recombination occurred during meiosis when the parasite was diploid. During meiosis, each progeny inherits on average half its chromosomes and associated *var* genes from each parent. If heterologous chromosomes only recombined at that stage, we should observe chimeric *var* sequences derived from *var* genes of different parents or from the same parent in an approximately 50∶50 ratio (Fig. 7). In contrast, we found that 26 out of 28 recombinations were ‘intra-strain’, i.e. involving pairs of *var* genes from the same parent. The simplest explanation is that these recombination events occurred during mitosis while the parental strains were in culture prior to the cross.

Finally, knowing the average mitotic *var* recombination rate calculated using our clone trees allows us to test whether the number of *var* recombination events observed in the crosses progeny is consistent with the culturing time before and after the cross. In our clone trees and other experiments [9], the SNP mutation rate is similar across strains (see above). Thus, the number of SNP mutations in a subclone is directly proportional to the number of intraerythrocytic life cycles that occurred before the last cloning step. The number of days of culture can then be inferred from the number of SNP mutations observed. Based on the number of *de novo* SNPs identified in the crosses progeny and our mitotic SNP mutation rate, we calculated that the *P. falciparum* strains used for the crosses had undergone 124 and 133 days of *in vitro* culturing for 3D7xHB3 and HB3xDd2 respectively. i.e. there had been 124 and 133 days in culture from when the original isolates were first sub-cloned (from NF54 for 3D7, H1 for HB3, *etc*), to the day the crosses' progeny were cloned out. For 3D7xHB3 and HB3xDd2, these estimates could be validated by direct comparison with the actual known culturing data of the parental clones, provided by Tom Wellems (NIH) and Lisa Ranford-Cartwright (University of Glasgow). The total recorded culture time, from isolation to progeny sub-cloning, was 89.5 and 99.5 days for the 3D7 and HB3 parasites used in the 3D7xHB3 cross – an average of 94.5 days. The equivalent time for the HB3 and Dd2 parasites used in the HB3xDd2 cross was 89 and 138 days respectively – an average of 113.5 days. These actual dates are very similar to the dates prdicted by our mutation analysis, which suggested that the 3D7xHB3 and HB3xDd2 isolates used for the crosses had been cultured for 124 and 133 days, where the real culture times were reassuringly close at approximately 94.5 and 113.5 days respectively (S7 Table). If we assume that the average mitotic *var* recombination rate is 6.44×10^−4^ recombinations per life cycle (the mean from the recombination rates calculated in the 3D7, Dd2, W2 and HB3 clone trees), then we would expect to find about 0.34 *var* recombinations per progeny sample. Again, these predictions, which are based on purely mitotic recombination, are very similar to what we observe: 0.38, 0.20 and 0.59 in 3D7xHB3, HB3xDd2 and 7G8xGB4, respectively. Therefore, the number of *var* recombinations found in the crosses progeny is consistent with what is expected from the number of mitotic events that the parasites have gone through both before and after the cross. No additional input of recombination from meiosis is required to explain the data.

## Discussion

By analysing whole genome sequences from hundreds of subclones derived from asexually replicating parasites, we have generated a large dataset of mitotic mutations in the *P. falciparum* genome. We found that *var* genes of groups B and C undergo ectopic recombination at a rate which greatly exceeds that of structural variation in other parts of the *P. falciparum* genome, and is not dissimilar to the SNP mutation rate. Recombining *var* genes often have low levels of sequence homology, and a pair of *var* genes can recombine at different positions on different occasions, so the new *var* sequences generated by this process are extremely diverse. However, the recombinant sequence is invariably in frame and free of errors, and *var* gene domain architecture is preserved. This process has presumably evolved to generate antigenic diversity in *P. falciparum* during the course of a single infection to evade the human immune response.

Our SNP and structural variant detection method by whole-genome sequencing was validated by PCR, capillary sequencing or both (S2–S4 Table). Sander *et al* recently published an analysis of *var* gene recombination in progeny of 3D7xHB3 and HB3xDd2 [19]. They reported that recombination breakpoints are concentrated near low folding free energy DNA 50-mers, with a minimal required sequence identity of around 20 bp with 10% mismatch. Three out of the four progeny samples in which they identified *var* recombinations were available to us. We independently found the exact same *var* recombination breakpoint coordinates, and came to the same conclusion that crossover events take place within short regions of high homology.

Generating diversity by structured recombination of regional gene segments is somewhat analogous to the way in which the vertebrate immune system generates diversity by V(D)J recombination of immunoglobulin and T cell receptor genes. However, our data show that the molecular mechanism of *var* gene recombination is markedly different from that of V(D)J recombination in two respects. First, the site of recombination was not marked by any specific sequence motif equivalent to the 'Recombination Signal Sequence' that targets recombinases in V(D)J recombination (S15 Fig.), and we observed instances of the same pair of *var* genes recombining at different positions on separate occasions (S8 Fig.). Second, V(D)J recombination depends on repair of DNA double-strand breaks (DSBs) by non-homologous end joining (NHEJ), a molecular pathway that appears to be missing or atypical in *P. falciparum* [21], [22]. All the instances of ectopic recombination we observed were achieved without errors or indels, indicating that the mechanism of DSB repair is more similar to homologous recombination (HR) than NHEJ, as this is generally high fidelity whereas NHEJ is error-prone. It is interesting to note that, lacking a typical NHEJ system, *P. falciparum* is presumably reliant on the machinery of HR for DSB repair. This is somewhat paradoxical given that the parasite is haploid for the majority of its life cycle, including the entirety of the mitotic intraerythrocytic stages. It therefore lacks homologous chromosome copies to use as templates for HR-mediated DSB repair. These conditions may be conducive for non-allelic homologous recombination during mitosis, with non-allelic templates being employed in the absence of homologous chromosomes.

While the pattern of *var* gene recombination therefore differs from well-known methods for generating diversity, it is also strikingly different from conventional HR in which crossovers take place in a region of extremely high sequence homology (>92%), typically extending over hundreds of base pairs [23]. Without extensive homology as a cue for pairing sequences from different regions of the genome, some alternative mechanism must exist for co-localisation of *var* genes before initiation of the recombination-inducing lesion [24]. *Var* genes are located in more than 30 regions of the genome, i.e. in most subtelomeric regions and in internal regions of four chromosomes. *P. falciparum* telomeres are tethered to the nuclear periphery where they form several distinct clusters [10] and it has been demonstrated by FISH that both subtelomeric and internal *var* genes aggregate within these clusters [25]. This nuclear clustering is believed to play an important role in the transcriptional activation and silencing of *var* genes [25] and it might also be a mechanism of co-localising sequences from different regions of the genome prior to ectopic recombination [10].

Our genome-wide approach in 3D7 subclones identified structural variants in and around *var* genes, indicating that these regions are more recombinogenic. In these samples, we did not observe any recombination in other variant surface antigen families such as *rifin* or *stevor*, despite the fact that there are nearly three times as many *rifin* genes as *var* genes in the genome. The rate of *var* gene recombination differed depending on *var* gene Ups type and parasite strain. We found a lower rate of recombination in HB3 than in 3D7, Dd2 or W2 parasites. The HB3 genome does not appear to be unable to recombine *var* genes *per sec*, as we did identify a recombined exon 1 *var* gene in the 'parent' strain used for one of our two HB3 clone trees and all clonal descendants from that parent inherited the recombined *var* gene. We cannot use this example to measure the recombination rate because we do not know the time in culture prior to performing our first clonal dilution, but the observation indicates that mitotic *var* exon 1 recombination in HB3 does occur, although perhaps at a rate that is too slow to be detected over the course of our clone trees. We have also observed instances of *var* recombinations in genetically modified HB3 lines (Hamilton *et al*, manuscript in preparation). A longer time in culture between clonal dilutions, or more clones per dilution, or both, would be necessary to capture enough *var* recombination events in this strain to determine its rate accurately. What factors influence *var* gene recombinogenicity, and whether there is a connection with drug resistance, remains to be determined. It is possible that HB3 is inherently less recombinogenic than other strains, or that the particular HB3 isolate we used in our clone experiments had, for unknown reasons, lost or reduced its capacity to undergo *var* recombination. Moreover, although *var* gene recombination in recently adapted *P. falciparum* field isolates does occur (Hamilton *et al*, manuscript in preparation), the frequency of such events in the wild and *in vivo* is currently unknown.

The fact that recombination was more common in group B and C *var* genes is consistent with the observation that group A *vars* from geographically diverse strains are more conserved than those of groups B and C [26]. *Var* recombination group-specificity also has implications for the underlying mechanism, raising the possibility that the upstream sequence or some other group-specific sequence feature plays a role in co-localising *var* genes prior to recombination. *Var* genes can also be classified into six groups based on their DBLα sequences [27]. However there was no association between *var* recombination and that specific subgrouping (Chi-square, p>0.40). Whether some specific *var* genes are inherently more recombinogenic than others, as the *Dd2var34*/*var45* example could suggest, requires an even larger dataset, perhaps involving several years of clone tree evolution, to properly assess. Further studies such as these will also be required to test the hypothesis that specific *var* genes being actively expressed in a given strain are more likely to recombine. If the latter hypothesis is true, it would partly explain the lack of recombination in group A genes that we observed, as cultured lab strains typically express very few, if any, of that subgroup [28]. It would also fit with the hypothesis that longer and more conserved *var* genes show a lower activation rate and lower sequence diversity [29]. Further investigation into DNA repair and recombination in *P. falciparum* will be essential to identify the precise molecular mediators of *var* recombination and elucidate the mechanism in full.

To date, *var* gene diversity has typically been studied using short DBLα tag sequences from field isolates [30], [31], or from 7 sequenced lab strains [8], [26], [32]. In either case, these *var* sequences represent snapshots of diversity at a particular time and place. They reflect the polymorphism found in the wild, under the intense pressure of the human immune system. Our study uses a novel approach to analyse *var* gene sequences as they are being generated, i.e. without any immunological selection. However, it has to be noted that lethal recombinations, for example between internal and subtelomeric *var* genes, would never be observed due to the resulting loss of genetic material. But only selection-free experiments such as this one can accurately measure the *var* recombination rate and the diversity of chimeric sequences being generated. As *var* gene assemblies from worldwide sampling will become available (Otto *et al*, manuscript in preparation), it will be of major interest to compare the sequence diversity under selection versus our dataset, for example to differentiate true recombination hotspots from selection hotspots. This will also help modelling studies to define and test recombinational constraints [32].

Comparison with our clone tree data suggests that much of the *var* gene recombination observed in the progeny of experimental genetic crosses likely arose during mitosis prior to the cross itself (Fig. 7). The dominance of mitotic recombination is inherent to the fact that the life cycle of *P. falciparum* consists of a single meiotic event for dozens or hundreds of mitoses. We propose a model whereby *var* gene sequence polymorphism is mainly generated during the asexual part of the life cycle, while meiosis has the crucial function of creating a new repertoire of ∼60 *var* genes from two different parents. There is also a clear biological advantage in having extensive recombination in antigen-coding genes take place during mitosis: it allows the continuous generation of antigenic diversity inside a human host over the course of a single infection. An adult with malaria fever typically carries on the order of 10^7^ to 10^10^ infected erythrocytes (parasitaemia 5 to 5,000 parasites/µl), so this implies that on the order of 10^4^ to 10^8^ new mosaic *var* gene sequences could be generated through recombination every two days in a single infected individual. *Var* gene expression is a mechanism of immune evasion, enabling parasitized erythrocytes to sequester in small blood vessels and thus avoid circulation through the spleen. But *var* genes are themselves the targets of host immunity as they elicit a strong antibody response, which the parasite evades by switching expression between different *var* genes. Ectopic recombination of *var* genes may be of limited value in the initial phase of infection, as each new *var* sequence is present in only a tiny fraction of parasites. However it could be of great importance in chronic asymptomatic infection, which is increasingly recognized a crucial problem for malaria elimination [33]. As infection progresses, the parasite population carried by a single individual has the potential to accumulate an almost limitless repertoire of antigenic variants, thus allowing the evolutionary selection of variants which are best equipped to evade the immune response in that particular individual over a protracted period of time.

## Materials and Methods

### *P. falciparum* culture and clone tree generation

Our 3D7 ‘parent’ clone was provided by Bob Pinches. It was one of several 3D7 reference stabilates produced in 1989 following the isolation of 3D7 from NF-54. Thus, its genome should be very close to the canonical 3D7 reference genome. Dd2, W2 and HB3 parasites were obtained originally from the Malaria Research and Reference Reagent Resource Center (MR4). All *P. falciparum* strains were cultured in human O+ erythrocytes with heat-inactivated 10% pooled human serum as in [34]. For the clonal dilution step, parasites were ultra-diluted into a 96-well plate, to reach a theoretical concentration of 0.2 to 0.5 parasites per well, supplemented with 100 µl of complete medium at 2% haematocrit. The chamber was gassed every 2–4 days with medium changes on day 4, 12 and 20 and 50% dilution with fresh 2%hct medium on day 8, 16 and 24. Positive wells were identified by medium colour change confirmed microscopically after day 16–24 post clonal dilution. The clones were further cultured in either 6-well plates or 10 ml flasks at 4–5%hct. Cultures were pelleted, mixed with RPMI to a total volume of 2 ml and frozen at −80°C for DNA extraction (see below), with most clones also being frozen in glycerolyte for reculture. After 1–2 months growth, one clone was arbitrarily selected for the next round of clonal dilution.

### DNA extraction, quantification and whole-genome sequencing

DNA extraction was performed with Qiagen whole blood midi kits [12145] following the manufacturer's instructions. DNA was quantified using Qubit 2.0 Fluorometer (Invitrogen, Carlsbad, CA, USA) according to manufacturer's instructions, with 1 µl from the extraction step. An average of 2 µg DNA (range 1–5 µg) per sample was submitted in 100 µl volume for PCR-free library preparation for next generation sequencing on the Illumina HiSeq, as described in [35], except without the initial PCR amplification step.

### Mapping to reference genome

All whole-genome sequencing output samples (100 bp paired-end reads) were processed through the MalariaGEN pipeline, as described in [35]. Briefly, FASTQ files were mapped to the *P. falciparum* 3D7 reference genome (PlasmoDB version 5.5) using BWA with default parameters (See S1 Table for coverage of each sample). For HB3, Dd2 and W2, the resulting BAM files were also mapped to the HB3 and Dd2 genomes from BROAD (assembled in 1187 and 2837 supercontigs, respectively) and to a Dd2 assembly made following the PAGIT protocol [36] (14 chromosomes +636 contigs, by T. Otto, unpublished data). When an *in silico* control was needed, these reference genomes were modified according to the predicted chromosomal rearrangements. For the discovery of translocations within *var* genes, samples were also mapped to each *var* sequence from their respective strain (sequences from [8]). This allows the identification of potential translocations between *var* genes that are located on the same chromosome.

### SNP calling

SAMtools mpileup followed by BCFtools were used for SNP detection [37]. Parent samples were analysed with the default parameters except for the following changes: Extended BAQ computation, unlimited read depth, use Bayesian inference, “−P” option <0.9 (probability that the site is a variant). Progeny samples were analysed with the following parameters: Extended BAQ computation, unlimited read depth, use Bayesian inference. The strict filter for progeny samples directly removes false positives. As the downstream analysis only keeps *de novo* SNPs called in progeny but not in parent, the looser filter for parent samples set here is also directed to remove false positives. Each progeny VCF file was then parsed using R with the following criteria: variant Quality >120, mapping quality >25, reject bi- or tri-allelic calls, at least 5 non-ref reads, number ref reads less than 20% of all reads. From this output list, each potential SNP was then visualised on LookSeq [38], an online tool in which sequence coverage is represented by a pile-up of blue reads matching the 3D7 reference genome, with mismatches in red. Finally, 95% of all detected SNPs had maximal quality (222) and a mapping quality >54. Note that a “progeny” sample was also analysed as a “parent” if that sample was subcloned.

### Detection of structural variants

In order to detect translocations involving *var* genes located on the same chromosome, reads from each sample were mapped with BWA to separate *var* gene sequences, i.e. each *var* sequence is considered its own “chromosome”.

DELLY identifies structural variation (>150 bp) by integrated paired-end and split-read analysis [39]. Paired ends mapping to loci with an increased or reduced spacer distance relative to what is expected based on insert size suggests either a deletion or an insertion, respectively. Face-away reads with increased spacer distance and higher coverage is suggestive of a duplication, and reads mapping to different chromosomes (‘trans-locus reads’) indicate a translocation (or recombination). DELLY was run on each 3D7-mapped BAM file, as well as all *var* gene mapped BAM files, with default parameters. For deletions and duplications, outputs were parsed to a minimum of 20 reads and a maximum distance of 50,000. For translocations, outputs were parsed to a minimum of 10 reads. Similar to SNP detection, non-specific hits were discarded by detecting structural variants in progeny that were also identified in parents. All resulting hits were inspected on LookSeq.

Progeny clones from the *P. falciparum* crosses 3D7xHB3 [18] (16 samples +2 parents), 7G8xGB4 [16] (27 samples +2 parents) and HB3xDd2 [17] (30 samples +2 parents) were whole genome sequenced as part of another genetic study (Miles, Pearson *et al*, manuscript in preparation). These clones were also searched for *var* gene translocations.

### Validating SNPs and structural variants

SNPs were validated by PCR amplifying a 150–800 bp fragment containing the putative SNP and capillary sequencing the PCR product (S6 Table).

Chimeric *var* genes identified by DELLY were validated through PCR, by designing primers that bridge the putative translocation site and only amplify product in samples possessing that translocation (S6 Table). The chimeric PCR product was then sub-cloned into a pCR Blunt-end Invitrogen vector backbone and 1–3 positive clones were capillary sequenced.

Quantitative real time PCR (qPCR) was used to validate copy number variations (CNV) in *var* genes. Primers were designed flanking the putative translocation site in the *PF3D7_0421100-0421300* chimera on chromosome 4. Assay was conducted in triplicate on a 96 well plate using a Roche Light Cycler 480 real-time PCR system. We estimated gene copy number relative to wild type 3D7, and normalized samples against the AMA1 gene (which has one copy in 3D7). Conditions as described in [40], [41].

### Calculating the SNP mutation and *var* exon 1 recombination rates

We estimated the SNP mutation rate per life cycle for each generation in the 3D7, Dd2 and HB3 clone trees, based on the number of SNPs identified per clone within that generation, and the number of life cycles since the previous clonal dilution:
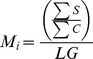



Where 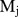 is the SNP mutation rate per life cycle for that clonal dilution generation, ∑S is the total number of SNPs identified for all the clones in that clonal dilution generation, ∑C is the total number of clones that were analysed in that clonal dilution generation, L is the number of life cycles that took place between the previous clonal dilution and the current one (see below), and G is the Genome size, taken as 23.3×10^6^ bp [42]. The number of life cycles was calculated as follows:
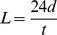



Where d is the days in culture between clonal dilutions, 24 to convert to hours, and t is the estimated life cycle time in hours. t has been measured in HB3 and Dd2 at 49.7 and 44.1 hours, respectively [43]. 48 hours was used for 3D7.

We took into account the number of clones in each clonal dilution generation to produce a weighted average mutation rate (S5 Table) of 4.07×10^−10^, 3.63×10^−10^ and 3.78×10^−10^ SNPs per erythrocytic life cycle per nucleotide for 3D7, Dd2 and HB3 respectively. We did not calculate a SNP mutation rate in W2 as the clone tree was not as extensive and we did not feel there was enough data for an accurate calculation. We observed one instance of two SNPs occurring ‘back to back’, i.e. two consecutive nucleotides were mutated from the reference, and one occasion where a single nucleotide separated two SNPs. These were all counted as separate SNP mutations for the rate calculation, though it may be that they represent single ‘mutation events’ at a molecular level.

The Bopp *et al* [9] published mutation rates for 3D7 and Dd2 in the absence of drug selection was 1.7×10^−9^ and 3.2×10^−9^, respectively. However, they adjusted their raw data to account for deleterious non-synonymous SNPs that may have been eliminated by purifying selection before being identified. Using their methods, our raw mutation rates become 4.28×10^−9^, 3.82×10^−9^ and 3.98×10^−9^ SNPs per erythrocytic life cycle per nucleotide for 3D7, Dd2 and HB3 respectively. Thus, there is high consistency both between ours and Bopp *et al*'s SNP mutation rate estimates and between different *P. falciparum* lab strains.

Our calculation of exon 1 *var* gene recombination in the Dd2 clone tree was very similar to our SNP mutation method described above (S5 Table). Because we often observed multiple crossovers per recombining *var* gene pair, we calculated two recombination rates: First, we calculated the rate at which pairs of *var* genes recombine in exon 1 in Dd2 as 2.32×10^−3^ per life cycle, i.e. a pair of *var* genes will recombine in ∼0.2% of infected erythrocytes per life cycle. We did not calculate the rate per life cycle per nucleotide because our analysis was focused on *var* exon 1, so dividing by the total nuclear genome was not appropriate. We therefore also calculated ‘per life cycle’ rates for SNP mutations to enable comparison between *var* recombination and SNPs. Second, we calculated the rate of *var* exon 1 crossover events at 3.45×10^−3^ per life cycle. This is higher than the rate of recombining *var* pairs per life cycle because some pairs have >1 crossover.

## Supporting Information

S1 Fig Generating clone trees. (A) The 3D7 clone tree. Each box indicates a whole-genome sequenced clone. Sample 3D7_1o was thawed and clonally diluted following whole genome sequence analysis indicated the presence of *var* gene recombination, to confirm that the mutation was inherited by clonally-derived progeny. (B) The Dd2 clone tree: samples Dd2_(A)1a and Dd2_(B)1b were further clonally diluted for three generations forming two ‘branches’, referred to as the A and B branches. (C) and (D) show two HB3 clone trees started independently. Unlike all other clone trees, HB3 (c) was initiated from a subclone of HB3. (E) The W2 clone tree. Asterisks on the x-axes indicate when clonal dilutions were performed.
(PDF)

S2 Fig Chimeric *var* gene created by recombination. This is the same example as simplified in Fig. 3A in the main manuscript. The translocations in clone 3D7_1o involved *var* genes *PF3D7_0426000* (‘*var4*’, red) on chromosome 4, *PF3D7_0937800* (‘*var9*’, green) on chromosome 9, and *PF3D7_0300100* (‘*var3*’, blue) on chromosome 3, all located in subtelomeres. There is a 2x increase in coverage for the first part of *var4* exon 1 in sample 3D7_1o, with the ‘parent’ strain (3D7_P) shown for reference (A). The trans-locus read peak located where the coverage drops is mate-paired with reads mapping to *var9* (B). *Var9* has zero coverage for the first part of exon 1. The boundary where the coverage starts is identical to the trans-locus reads peak, with mate-paired reads from *var4*. The chimeric gene therefore had a duplicated first section of *var4* exon 1, joined with the remainder of *var9*. We validated this model by PCR amplifying and capillary sequencing a product bridging the var4/9 translocation site, with no other tested samples producing a band. The translocation was also inherited by all four progeny of 3D7_1o. (E). In addition, a second trans-locus read peak in *var9* was found in exon 2 (B), with mate pairs mapping to *var3* exon 2 (C). *Var9* lacks any coverage in exon 2, whilst *var3* has 2x exon 2 coverage. We thus suspect three translocation events in clone 3D7_1o, involving chromosomes 4, 9 and 3. The final arrangement left 3D7_1o with intact *var4* and *var9*, and a chimeric *var4-9-3* composed of *var4+9* for exon 1 with the exon 2 from *var3* (D). TLR =  Trans-locus reads.
(PDF)

S3 Fig Example of 7 recombination events within *var* gene exon2 in 3D7_1b. An example of multiple cross-overs in between the same two var gene is found in clone 3D7_1b, in which most of PF3D7_0300100 was deleted and replaced by a duplicated copy of PF3D7_1373500, as shown by TLR peaks, coverage and validated by PCR amplification. Capillary sequencing the PCR amplicon of the chimeric *var3/13* exon 2 revealed that the new exon 2 sequence switched back and forth between *var3* and *var13* seven times within a ∼700 bp distance. The shortest distance observed between recombination break points was only 54 bp, indicating an incredibly active cross-over recombination process. (A) The two upper panels show reads mapped to the exon 2 of *PF3D7_0300100* in 3D7_P and 3D7_1b. All other progeny showed the same mapping as the 3D7_P parent. (B) Primers that bridge the var3/13 translocation site (i.e. one annealing to var3, the other to var13) amplified a product in 3D7_1b but not in any other sub-clones tested. Note the fainter band in the 3D7 parent (3D7_P), meaning that the frequency of the var3/13 mutation in 3D7_P was high enough prior to sub-cloning for it to be detectable by PCR. The band from 3D7_1b was gel extracted, sub-cloned into the pCR Blunt-end Invitrogen vector backbone and 3 positive clones were capillary sequenced. This was repeated with an independent primer pair bridging the translocation site. (C) The capillary sequence of this entire exon 2 in 3D7_1b was used to reconstruct the model. The chimeric sequence swaps 7 times from *PF3D7_0300100* (red) to *PF3D7_1373500* (blue). Blocks where red and blue colours are merged (limits indicated by nucleotide coordinates) correspond to the Identity Blocks. Finally, the *var* exon 2 of PF3D7_0300100 was replaced by the capillary sequence in the reference genome. All reads from 3D7_135 were mapped to this updated reference genome (lower panel of Fig. A).
(PDF)

S4 Fig Triple recombination event within less than 50 bp. DELLY identified one translocation event between *PF3D7_1041300* and *PF3D7_0500100* in sample 3D7_1o. (B) is a simplified model of (A). Trans-locus reads on LookSeq are blue when they match the reference sequence and red when there is a mismatch. All mismatches near the DELLY-predicted recombination breakpoint actually correspond to the sequence from the other *var* that has been translocated. Mismatches and perfect matches indicate the following chimeric sequence from upstream to downstream: *PF3D7_1041300* sequence (blue), Identity Block of 11 bp, *PF3D7_0500100* sequence (1 bp), second identity block (11 bp), *PF3D7_1041300* sequence (1 bp), third identity block (27 bp), *PF3D7_0500100* sequence (red). The capillary sequence from sample 3D7_1o confirms this model (although the capillary sequence does not cover the full alignment shown here, it does confirm the triple recombination events).
(PDF)

S5 Fig Duplications in internal *var* gene clusters can also generate chimeras. *Var* genes within the body of a chromosome are found in clusters of >4 on chromosomes 4, 8 and 12 in 3D7, and all face the same direction. ‘Duplication-chimeras’ in our clone tree were formed by duplicating the second half of one *var* gene and the first half of the *var* gene immediately adjacent, splicing the two halves together as a chimera. In this example (A), there is 2-fold increase coverage for the second part of *PF3D7_1240400* (red) and the first part of the adjacent gene, *PF3D7_1240600* (green), on chromosome 12 of all 3D7 samples. This generated a chimeric *var* gene, validated by PCR, capillary sequencing and transgenerational inheritance of the chimeric product (B). The chimeric gene was deleted in clone 3D7_2b, as shown by PCR and the loss of 2x coverage (C).
(PDF)

S6 Fig Another example of internal *var* gene clusters generating chimeras. The entire region in our 3D7 parent, containing (at least) four *var* genes, has double coverage (A), suggesting multiple duplications have occurred in our 3D7 parental strain relative to that used for compiling the 3D7 reference genome. On top of this baseline elevated coverage, clone 3D7_1a appeared to have a further duplication spanning the first half of exon 1 from *PF3D7_0421100* and the second half of exon 1 plus the intron and exon 2 from *PF3D7_0421300*. We predicted this would generate a chimeric *PF3D7_0421100-0421300* gene with the two halves of exon 1 linked. To our initial surprise, this model was confirmed by PCR (B) and capillary sequencing as being present in every sample checked, not just clone 3D7_1a. We therefore conjectured that this chimeric *var* gene was part of the baseline duplications causing elevated coverage here in all samples, but that clone 3D7_1a possessed >1 copy of the chimera. We tested this using quantitative real time PCR (qPCR) of genomic DNA, which confirmed that clone 3D7_1a possessed extra copies of the chimeric sequence (C), whereas three randomly selected control clones (3D7_1h, 3D7_1m and 3D7_1o) all had only one copy. The graph depicts the average 2^−CP value^ for the amplification product produced by primers flanking the *PF3D7_0421100* - *PF3D7_0421300* chimera crossover point. All CP values were normalized against amplification of the AMA1 gene, which has only one copy in the 3D7 genome, and then against sample 3D7_1h. The qPCR suggests that sample 3D7_1a has three copies of the *PF3D7_0421100-0421300* chimera. All qPCRs done in triplicate. Note that 'CP' is the value given by the Roche Light Cycler 480 real-time PCR system. Other qPCR machines use a 'CT' value. (See Methods for further details, and Supplementary S6 Table for primer sequences).
(PDF)

S7 Fig Evolution of a *var* gene. See also Fig. 2 in the main manuscript for a summary. (A) Coverage plots for *Dd2var34* and *Dd2var45* from LookSeq. The lower part shows trans-locus pair-end reads only. Two peaks are found in sample Dd2_(A)1a and all its progeny (not shown here). The coordinates of these trans-locus peaks correspond exactly to the rise in coverage for *Dd2var34* and to the fall in coverage in *Dd2var45*. (B) Model describing this chromosomal rearrangement: a new chimeric *var* gene has been created in sample Dd2_(A)1a, the sequence being an hybrid between *Dd2var34* and *Dd2var45*. (C) Trans-locus pair-end reads mapped to a reference genome where the *Dd2var34* sequence has been replaced by the chimera *Dd2var34-45-34*. Note that peaks in Dd2_(A)1a have now disappear. In two of its progeny, Dd2_(A)2g and Dd2_(A)4b, 3 and 1 new translocation peaks have appeared, respectively. (D) PCR with primers overlapping the translocated sequence. In *Dd2var34*, the primers give a 3974 bp product, which is seen in all clones. In the chimeric *var*, the primers give a smaller product of 2756 bp, seen only in sample Dd2_(A)1a and its progeny. As predicted, clone Dd2(A)4b has lost *Dd2var34* while clone DD2_(A)4 l has lost the chimera *Dd2var34-45-34*. * Unspecific single read peaks found in all samples.
(PDF)

S8 Fig All *var* exon 1 translocations identified in this study. Recombination breakpoints are marked by a vertical back line, and linked with the recombining *var*. The data for this figure can be found in S4 Table. For simplicity, only homology blocks 1 to 10 are represented here, with small colour rectangles above each domain. In two instances, the coverage of trans-locus reads did not allow the identification of the recombination breakpoint coordinates (as it is normally done in S4 Fig. for example), hence the lack of black lines between GB4var21-GB4var43, and GB4var128-GB4var21. NA =  unknown chromosomal location.
(TIF)

S9 Fig Group B/C *var* genes do not necessarily recombine with var genes of highest similarity. *Var* genes were pairwise aligned within each strain. Percentage of nucleotide identity of the recombining var is shown in red. Group A and E var genes, where no recombination had been observed in our dataset, are shown in dark blue. Nucleotide identities between group B/C *var* genes ranged from 53 to 99% (average  = 66.8%). The two recombining exon 1 *var* genes had an average of 63% Identity (range 42 to 73%). Data from the 7G8 and GB4 strains is not represented here as their *var* genes are not fully assembled.
(TIF)

S10 Fig Defining identity blocks. Var B/C genes recombine (A) ClustalW alignment of *Dd2var34* and *Dd2var45* at a crossover. Example sequence reads mapped to *Dd2var34* and *Dd2var45* are shown above and below the alignment respectively. Sequence reads are coloured blue when they match the reference and red when there is a mismatch. All mismatches near the recombination breakpoint correspond to the sequence from the other *var* that has been translocated. The sequence between the mismatches is where the crossing-over event must have occurred, and is by definition identical between both *var* genes. We term these sequences ‘identity blocks’. (B) Cartoon demonstrating formation of ‘*Dd2var34/45*’ chimera, with recombination breakpoint occurring within the identity block.
(PDF)

S11 Fig Recombination in var genes occurs where sequences are similar. Dot plot matrix with each pair of recombining var genes indicate the length of each BLAST hit (black line). Location of recombination breakpoints are indicated by red crosses. BLAST was run using default parameters.
(TIF)

S12 Fig Var gene exon 1 recombine in short regions of higher homology. Each plot is a ClustalW2 DNA alignment (default parameters) of a pair of recombining var genes. The Y-axis shows the percentage of identities (overlapping 50 bp windows) between the two sequences. The red triangles indicate where the recombination breakpoint was found. Plots without any triangle mean that the precise breakpoint coordinate could not be identified.
(TIF)

S13 Fig Distribution of *var* exon 1 recombination within each domain class. Blue bars indicate the frequency of each domain, normalized by their length, relative to all domains of group B and C *var* genes from the 3D7, HB3 and Dd2 strains. Red bars show the frequency of recombinations observed within each domain class (data from S4 Table).
(EPS)

S14 Fig No recombination hotspot within DBL domains. Left panel: DBL domains are made of three subdomains (S1, S2 and S3). Homology blocks (HB) 1 to 5 are also shown, with the 100 bp upstream sequence in red. Right panel: The number of DBL recombination breakpoints within HB 1 to 5 (blue) and in the 100 bp upstream of these HB (red) is plotted. Rask *et al* postulated the existence of a recombination hotspot at the S2-S3 boundary of the DBL domain, i.e. upstream and within HB2, due to high sequence polymorphism found in seven P. falciparum genomes (Rask et al. 2010). Because there is no immune pressure *in vitro*, our experiment allows us to differentiate underlying recombination rate from the effects of natural selection. We found no evidence of a recombination hotspot in Rask *et al*'s locus, suggesting that parasites with recombination breakpoints here are being favoured by natural selection, rather than an increased recombination rate per se.
(EPS)

S15 Fig No conserved motifs within Identity Blocks. (A) ClustalW alignment of 71 Identity Blocks. (B) LOGO representation of the most conserved nucleotides. Note that the ‘A’ and ‘T’ at positions 6, 12, 62 and 67 represent only two identity block sequences, i.e. they are not particularly conserved. (C) Top 3 most common motifs identified by MEME, using Identity Blocks ≥8 bp.
(PDF)

S1 Table Genome coverage for each subclone.
(XLSX)

S2 Table SNPs identified in clone trees.
(XLSX)

S3 Table Structural variants identified in the 3D7 clone tree, represented in Fig. 1.
(XLSX)

S4 Table All *var* gene recombinations.
(XLSX)

S5 Table Mitotic point mutation rates and *var* gene recombination rates measured from clone trees.
(XLSX)

S6 Table Primers used for validating point mutations and *var* gene recombination by PCR amplification and capillary sequencing.
(XLSX)

S7 Table Crosses progeny culture time and *var* gene recombinations.
(XLSX)
