# Supplementary figures and images for: Generation of Antigenic Diversity in Plasmodium falciparum by Structured Rearrangement of Var Genes During Mitosis
Source: PLoS Genet. 2014 Dec 18;10(12):e1004812. doi: 10.1371/journal.pgen.1004812 (PMC4270465; doi:10.1371/journal.pgen.1004812)

A

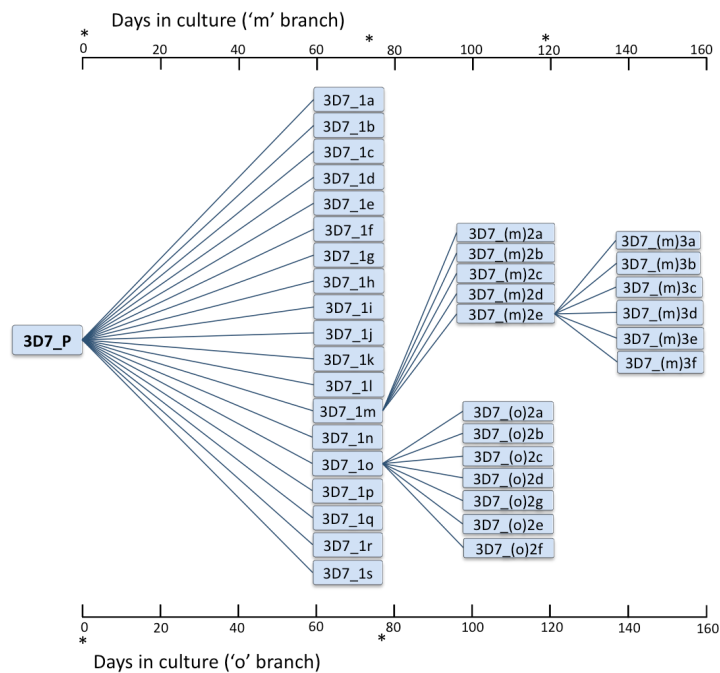

B

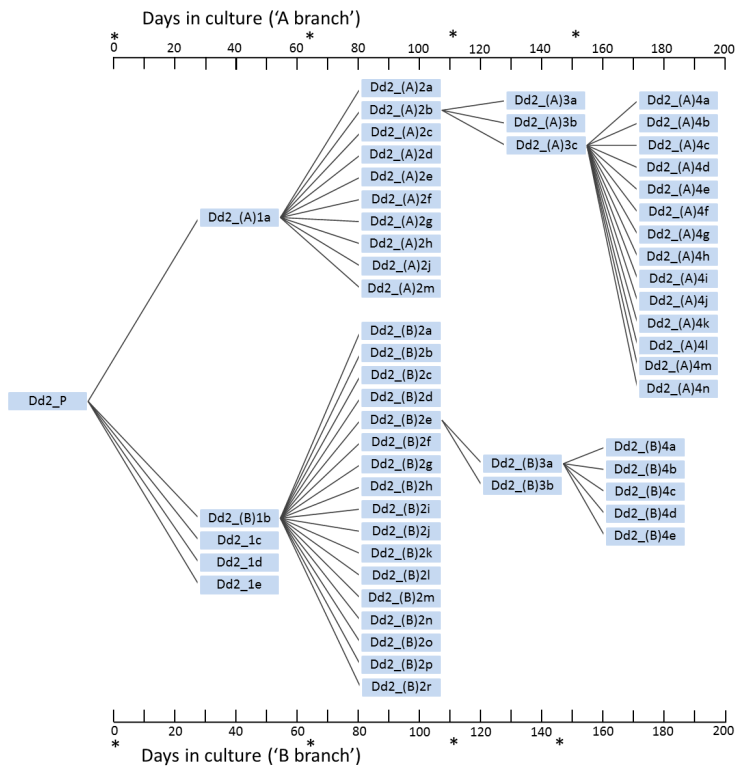

C

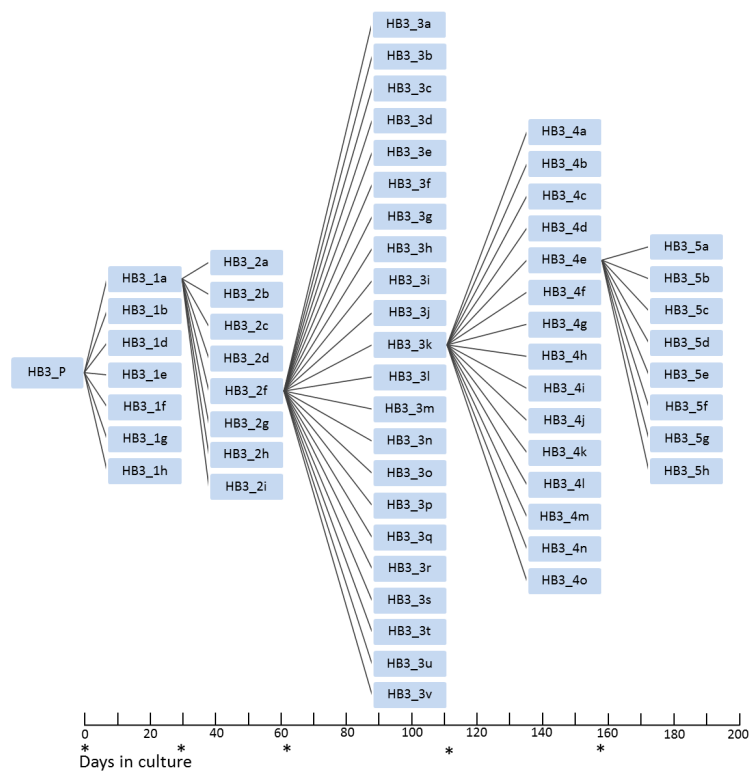

D

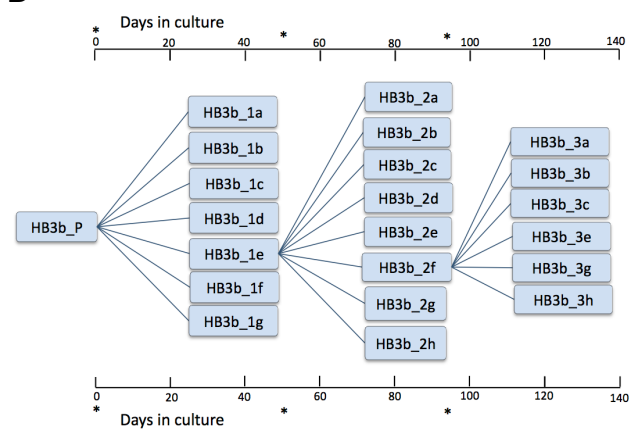

E

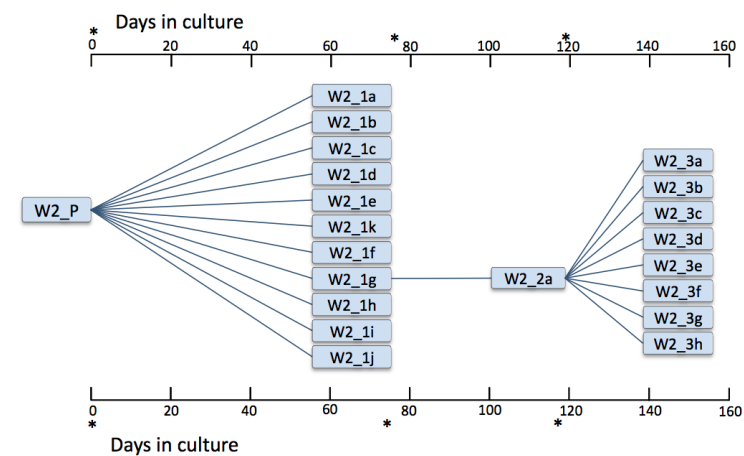

Supplement: S1 Fig — Generating clone trees. (A) The 3D7 clone tree. Each box indicates a whole-genome sequenced clone. Sample 3D7_1o was thawed and clonally diluted following whole genome sequence analysis indicated the presence of var gene recombination, to confirm that the mutation was inherited by clonally-derived progeny. (B) The Dd2 clone tree: samples Dd2_(A)1a and Dd2_(B)1b were further clonally diluted for three generations forming two ‘branches’, referred to as the A and B branches. (C) and (D) show two HB3 clone trees started independently. Unlike all other clone trees, HB3 (c) was initiated from a subclone of HB3. (E) The W2 clone tree. Asterisks on the x-axes indicate when clonal dilutions were performed. (PDF) [file pgen.1004812.s001.pdf]

**A**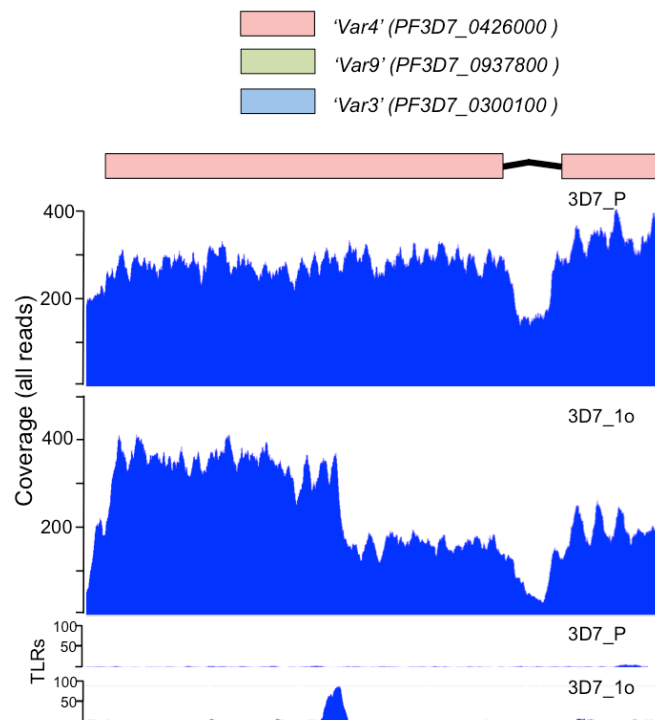**B**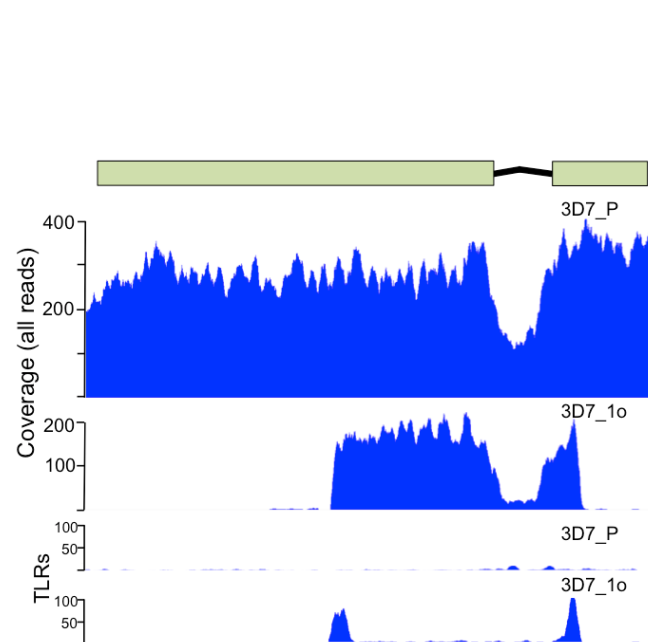**C**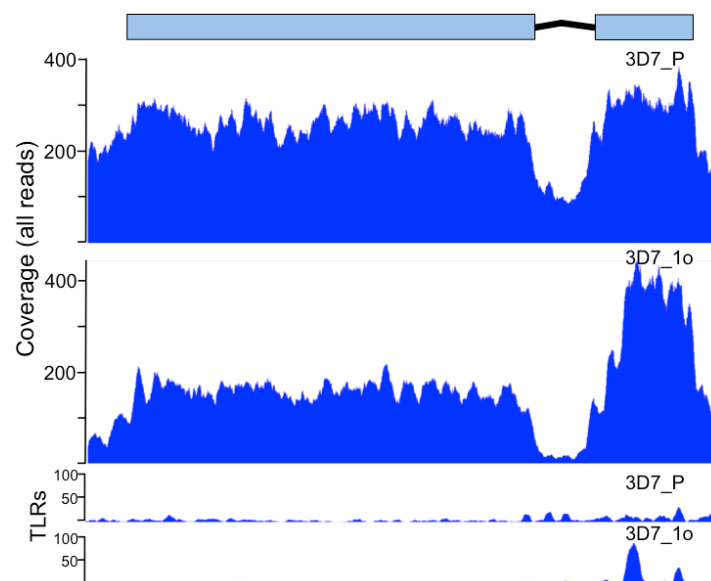**D**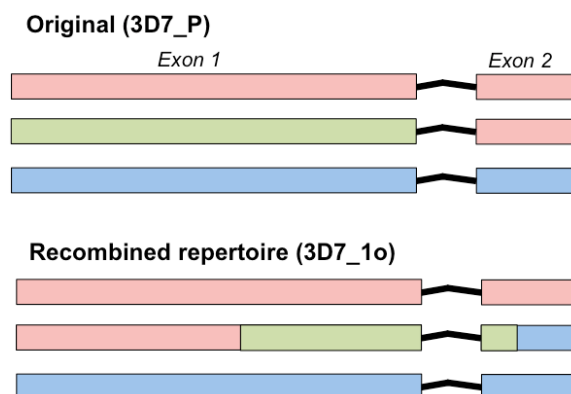**E**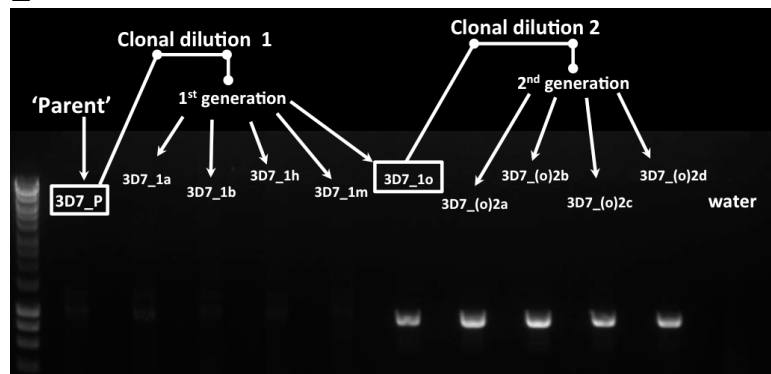

Supplement: S2 Fig — Chimeric var gene created by recombination. This is the same example as simplified in Fig. 3A in the main manuscript. The translocations in clone 3D7_1o involved var genes PF3D7_0426000 (‘var4’, red) on chromosome 4, PF3D7_0937800 (‘var9’, green) on chromosome 9, and PF3D7_0300100 (‘var3’, blue) on chromosome 3, all located in subtelomeres. There is a 2x increase in coverage for the first part of var4 exon 1 in sample 3D7_1o, with the ‘parent’ strain (3D7_P) shown for reference (A). The trans-locus read peak located where the coverage drops is mate-paired with reads mapping to var9 (B). Var9 has zero coverage for the first part of exon 1. The boundary where the coverage starts is identical to the trans-locus reads peak, with mate-paired reads from var4. The chimeric gene therefore had a duplicated first section of var4 exon 1, joined with the remainder of var9. We validated this model by PCR amplifying and capillary sequencing a product bridging the var4/9 translocation site, with no other tested samples producing a band. The translocation was also inherited by all four progeny of 3D7_1o. (E). In addition, a second trans-locus read peak in var9 was found in exon 2 (B), with mate pairs mapping to var3 exon 2 (C). Var9 lacks any coverage in exon 2, whilst var3 has 2x exon 2 coverage. We thus suspect three translocation events in clone 3D7_1o, involving chromosomes 4, 9 and 3. The final arrangement left 3D7_1o with intact var4 and var9, and a chimeric var4-9-3 composed of var4+9 for exon 1 with the exon 2 from var3 (D). TLR = Trans-locus reads. (PDF) [file pgen.1004812.s002.pdf]

**A**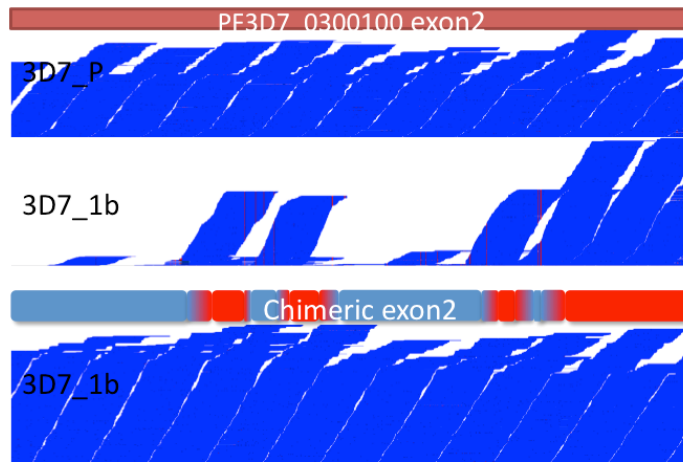**B**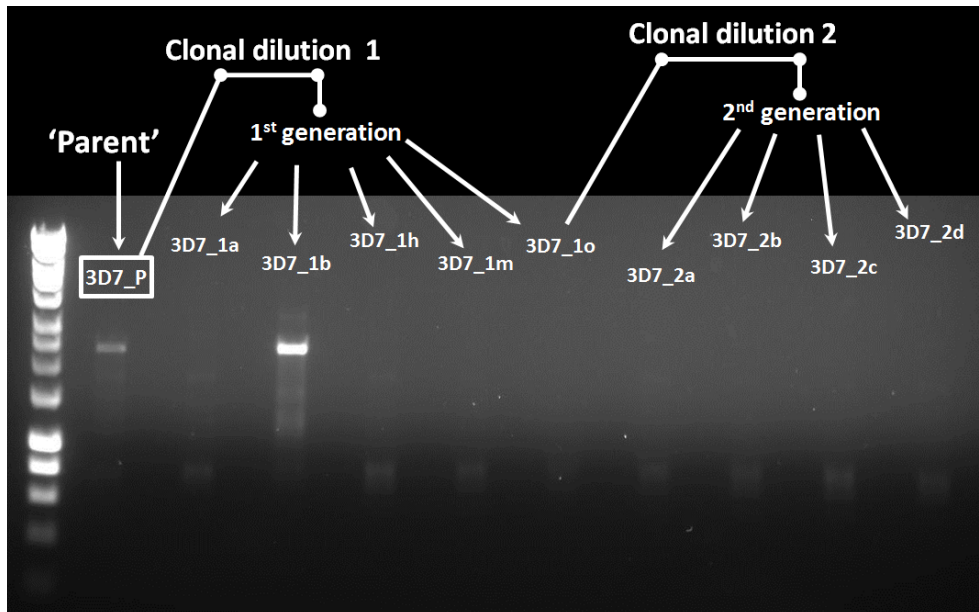**C**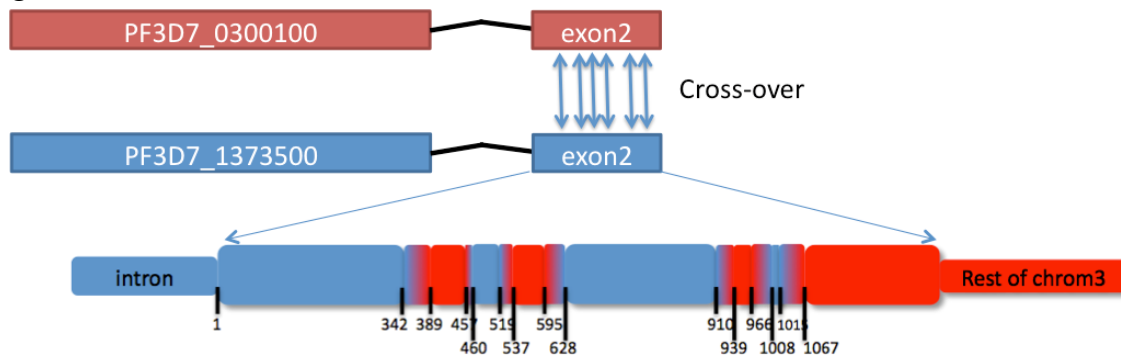

Supplement: S3 Fig — Example of 7 recombination events within var gene exon2 in 3D7_1b. An example of multiple cross-overs in between the same two var gene is found in clone 3D7_1b, in which most of PF3D7_0300100 was deleted and replaced by a duplicated copy of PF3D7_1373500, as shown by TLR peaks, coverage and validated by PCR amplification. Capillary sequencing the PCR amplicon of the chimeric var3/13 exon 2 revealed that the new exon 2 sequence switched back and forth between var3 and var13 seven times within a ∼700 bp distance. The shortest distance observed between recombination break points was only 54 bp, indicating an incredibly active cross-over recombination process. (A) The two upper panels show reads mapped to the exon 2 of PF3D7_0300100 in 3D7_P and 3D7_1b. All other progeny showed the same mapping as the 3D7_P parent. (B) Primers that bridge the var3/13 translocation site (i.e. one annealing to var3, the other to var13) amplified a product in 3D7_1b but not in any other sub-clones tested. Note the fainter band in the 3D7 parent (3D7_P), meaning that the frequency of the var3/13 mutation in 3D7_P was high enough prior to sub-cloning for it to be detectable by PCR. The band from 3D7_1b was gel extracted, sub-cloned into the pCR Blunt-end Invitrogen vector backbone and 3 positive clones were capillary sequenced. This was repeated with an independent primer pair bridging the translocation site. (C) The capillary sequence of this entire exon 2 in 3D7_1b was used to reconstruct the model. The chimeric sequence swaps 7 times from PF3D7_0300100 (red) to PF3D7_1373500 (blue). Blocks where red and blue colours are merged (limits indicated by nucleotide coordinates) correspond to the Identity Blocks. Finally, the var exon 2 of PF3D7_0300100 was replaced by the capillary sequence in the reference genome. All reads from 3D7_135 were mapped to this updated reference genome (lower panel of Fig. A). (PDF) [file pgen.1004812.s003.pdf]

**A**

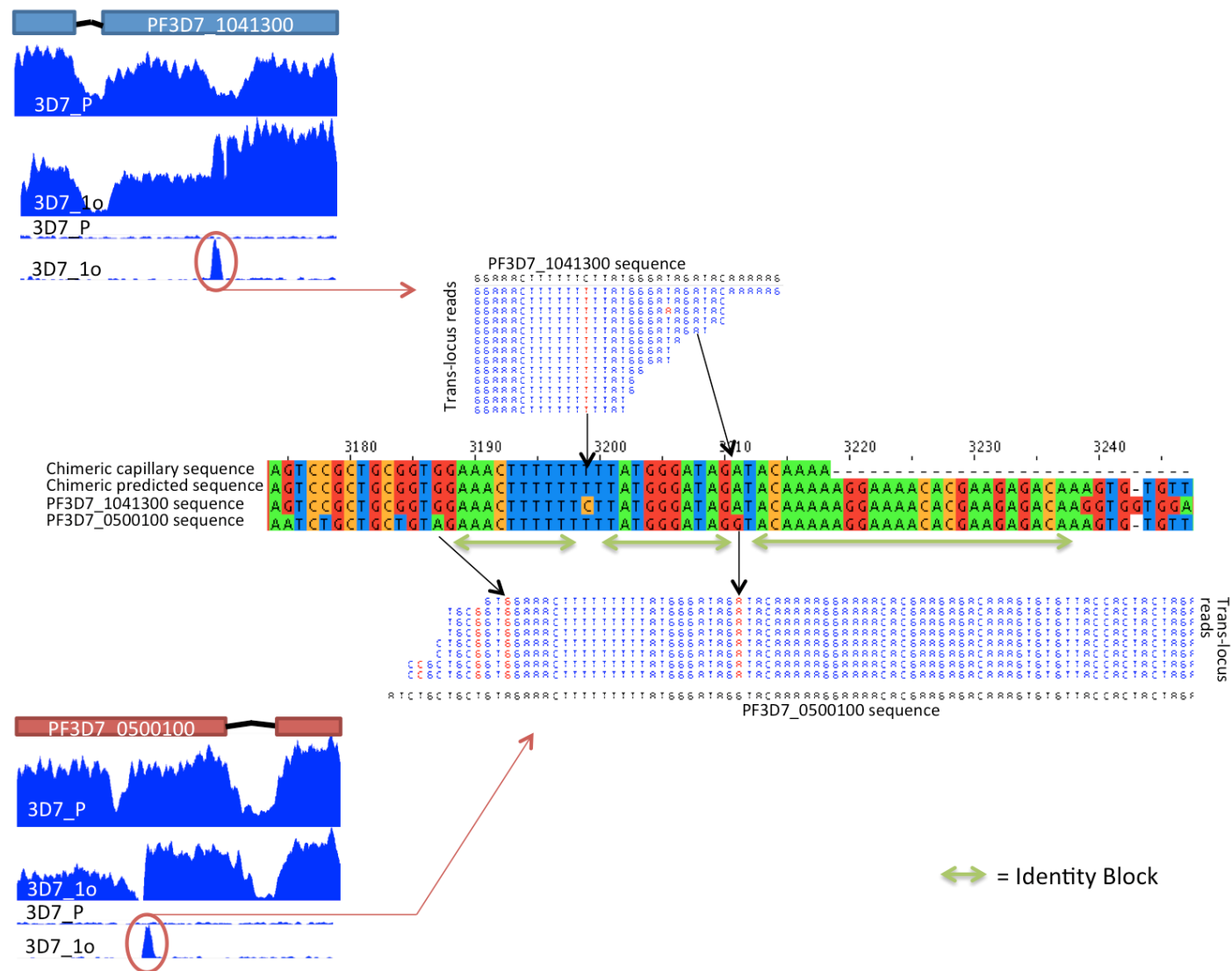

**B**

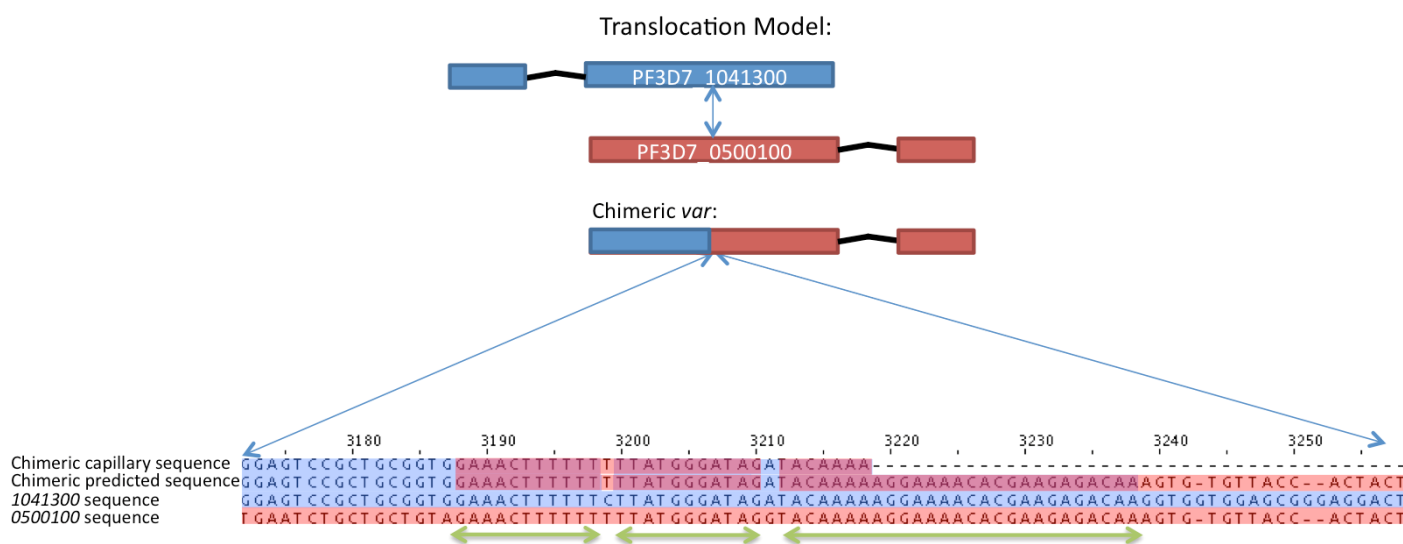

Supplement: S4 Fig — Triple recombination event within less than 50 bp. DELLY identified one translocation event between PF3D7_1041300 and PF3D7_0500100 in sample 3D7_1o. (B) is a simplified model of (A). Trans-locus reads on LookSeq are blue when they match the reference sequence and red when there is a mismatch. All mismatches near the DELLY-predicted recombination breakpoint actually correspond to the sequence from the other var that has been translocated. Mismatches and perfect matches indicate the following chimeric sequence from upstream to downstream: PF3D7_1041300 sequence (blue), Identity Block of 11 bp, PF3D7_0500100 sequence (1 bp), second identity block (11 bp), PF3D7_1041300 sequence (1 bp), third identity block (27 bp), PF3D7_0500100 sequence (red). The capillary sequence from sample 3D7_1o confirms this model (although the capillary sequence does not cover the full alignment shown here, it does confirm the triple recombination events). (PDF) [file pgen.1004812.s004.pdf]

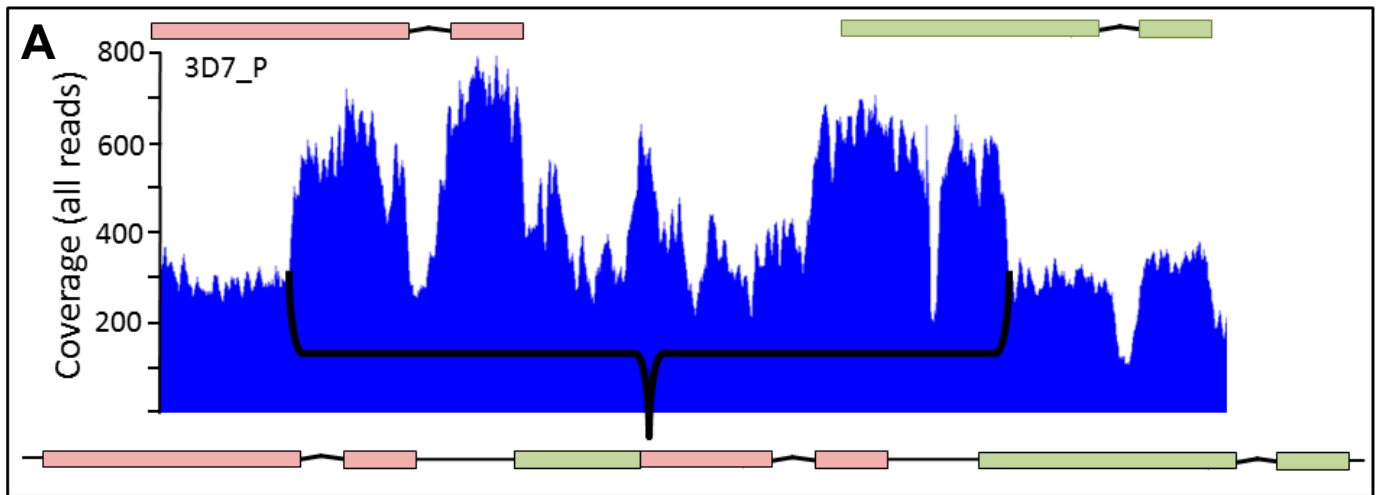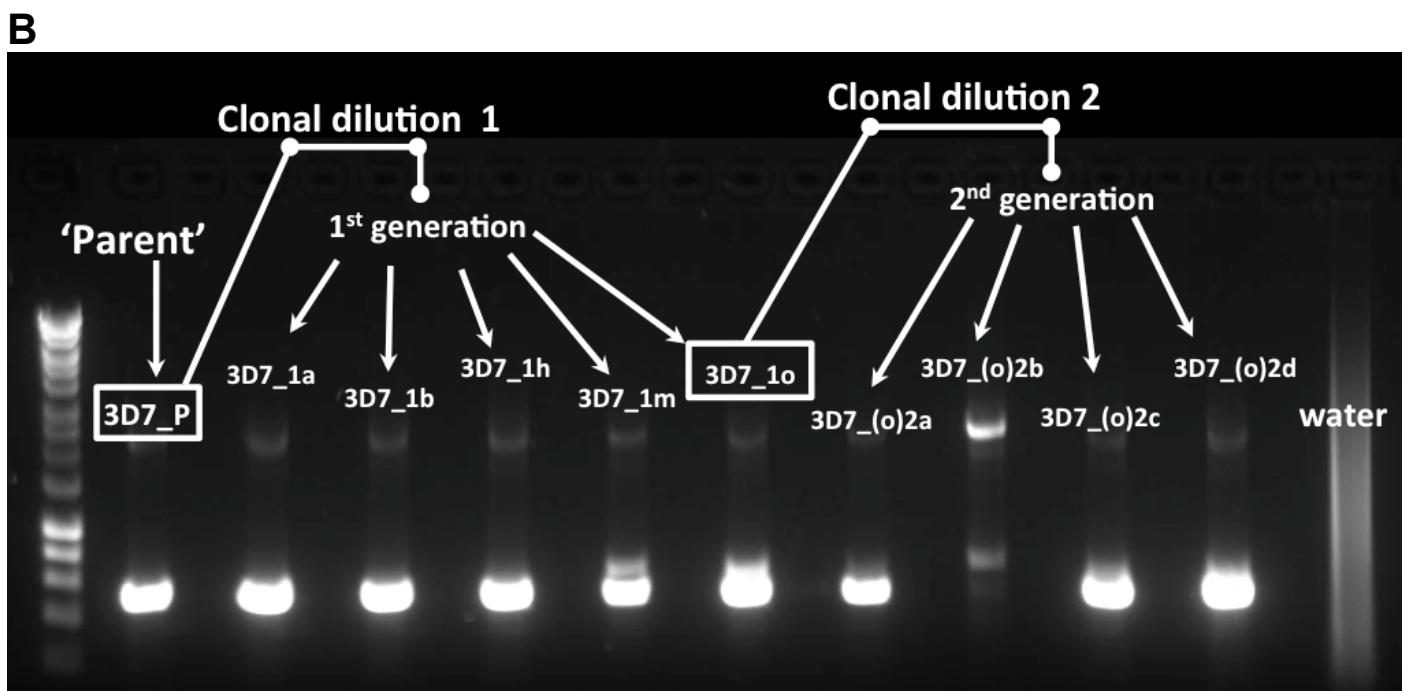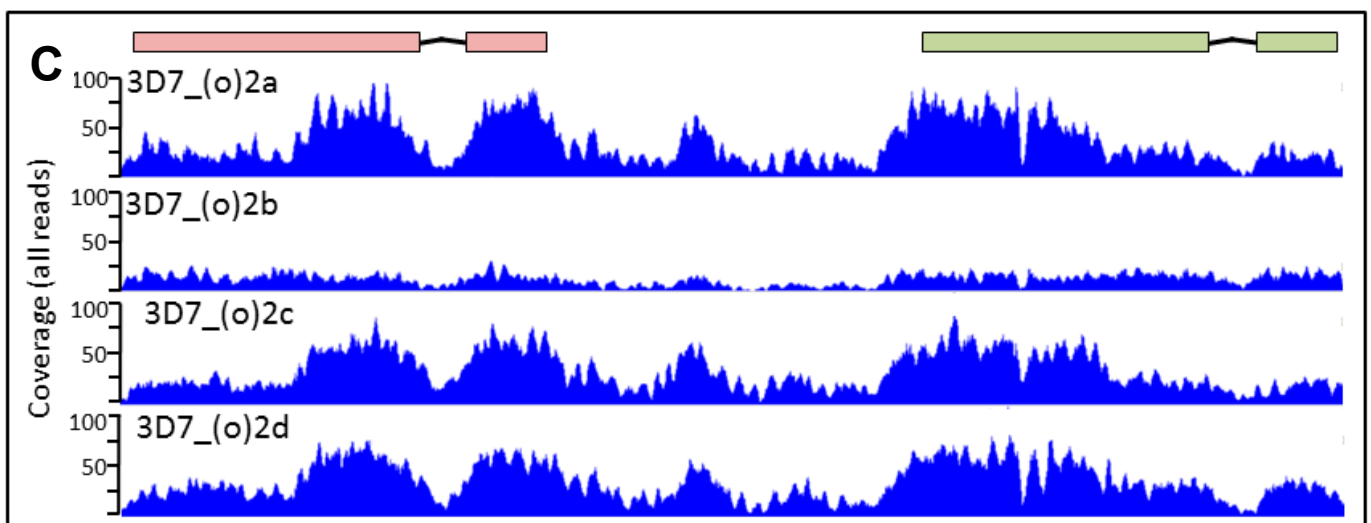

Supplement: S5 Fig — Duplications in internal var gene clusters can also generate chimeras. Var genes within the body of a chromosome are found in clusters of >4 on chromosomes 4, 8 and 12 in 3D7, and all face the same direction. ‘Duplication-chimeras’ in our clone tree were formed by duplicating the second half of one var gene and the first half of the var gene immediately adjacent, splicing the two halves together as a chimera. In this example (A), there is 2-fold increase coverage for the second part of PF3D7_1240400 (red) and the first part of the adjacent gene, PF3D7_1240600 (green), on chromosome 12 of all 3D7 samples. This generated a chimeric var gene, validated by PCR, capillary sequencing and transgenerational inheritance of the chimeric product (B). The chimeric gene was deleted in clone 3D7_2b, as shown by PCR and the loss of 2x coverage (C). (PDF) [file pgen.1004812.s005.pdf]

**A**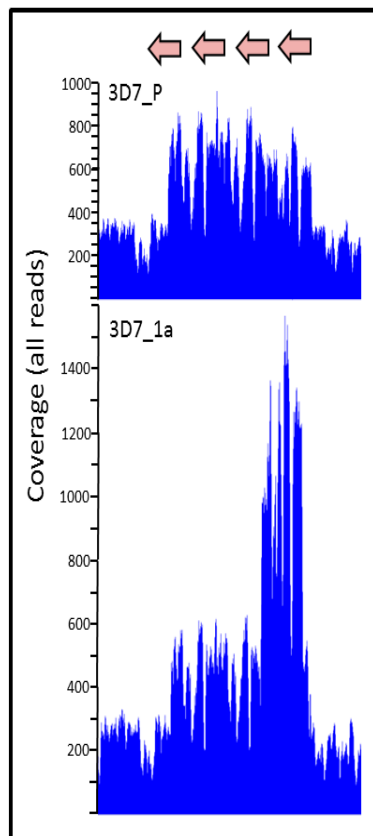**B**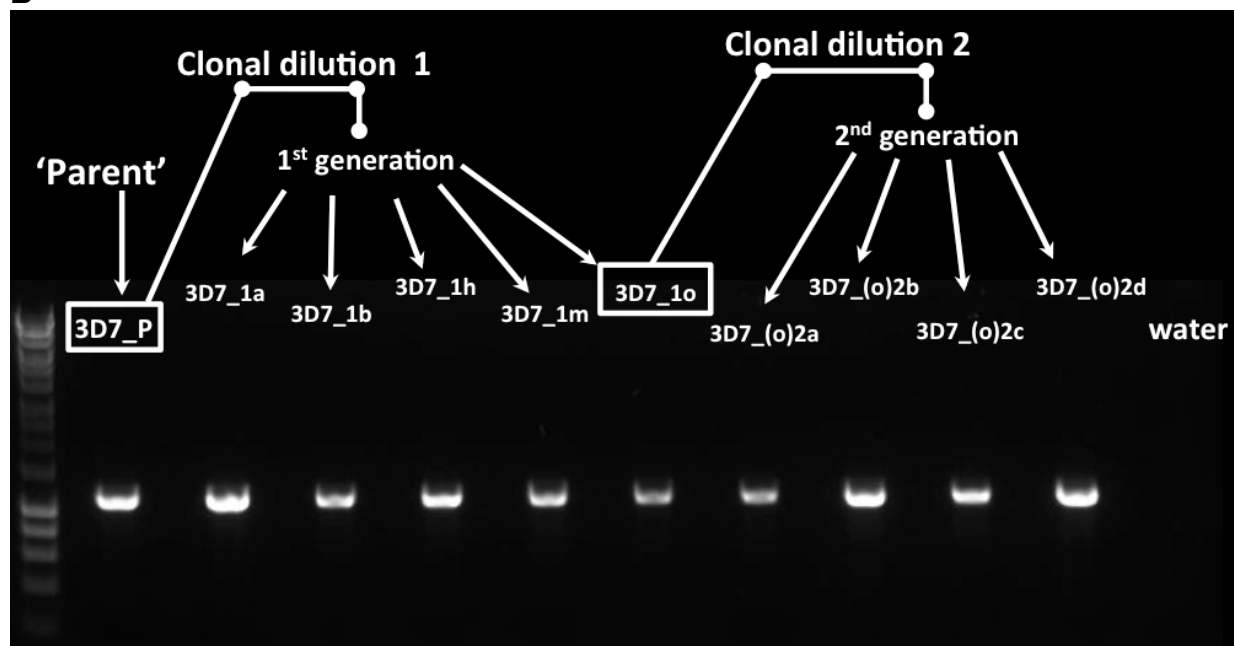**C**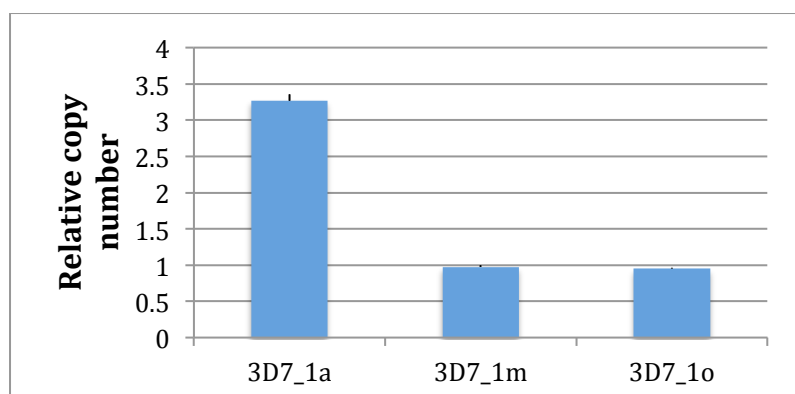

Supplement: S6 Fig — Another example of internal var gene clusters generating chimeras. The entire region in our 3D7 parent, containing (at least) four var genes, has double coverage (A), suggesting multiple duplications have occurred in our 3D7 parental strain relative to that used for compiling the 3D7 reference genome. On top of this baseline elevated coverage, clone 3D7_1a appeared to have a further duplication spanning the first half of exon 1 from PF3D7_0421100 and the second half of exon 1 plus the intron and exon 2 from PF3D7_0421300. We predicted this would generate a chimeric PF3D7_0421100-0421300 gene with the two halves of exon 1 linked. To our initial surprise, this model was confirmed by PCR (B) and capillary sequencing as being present in every sample checked, not just clone 3D7_1a. We therefore conjectured that this chimeric var gene was part of the baseline duplications causing elevated coverage here in all samples, but that clone 3D7_1a possessed >1 copy of the chimera. We tested this using quantitative real time PCR (qPCR) of genomic DNA, which confirmed that clone 3D7_1a possessed extra copies of the chimeric sequence (C), whereas three randomly selected control clones (3D7_1h, 3D7_1m and 3D7_1o) all had only one copy. The graph depicts the average 2−CP value for the amplification product produced by primers flanking the PF3D7_0421100 - PF3D7_0421300 chimera crossover point. All CP values were normalized against amplification of the AMA1 gene, which has only one copy in the 3D7 genome, and then against sample 3D7_1h. The qPCR suggests that sample 3D7_1a has three copies of the PF3D7_0421100-0421300 chimera. All qPCRs done in triplicate. Note that 'CP' is the value given by the Roche Light Cycler 480 real-time PCR system. Other qPCR machines use a 'CT' value. (See Methods for further details, and Supplementary S6 Table for primer sequences). (PDF) [file pgen.1004812.s006.pdf]

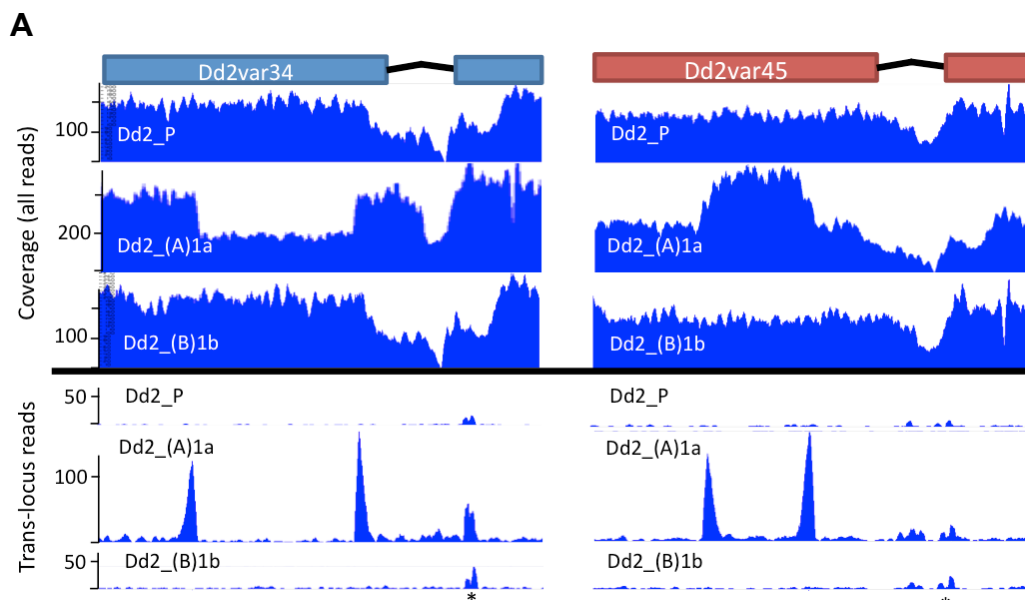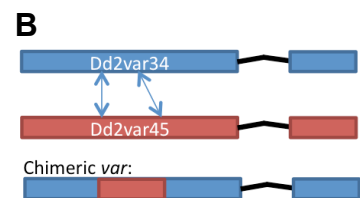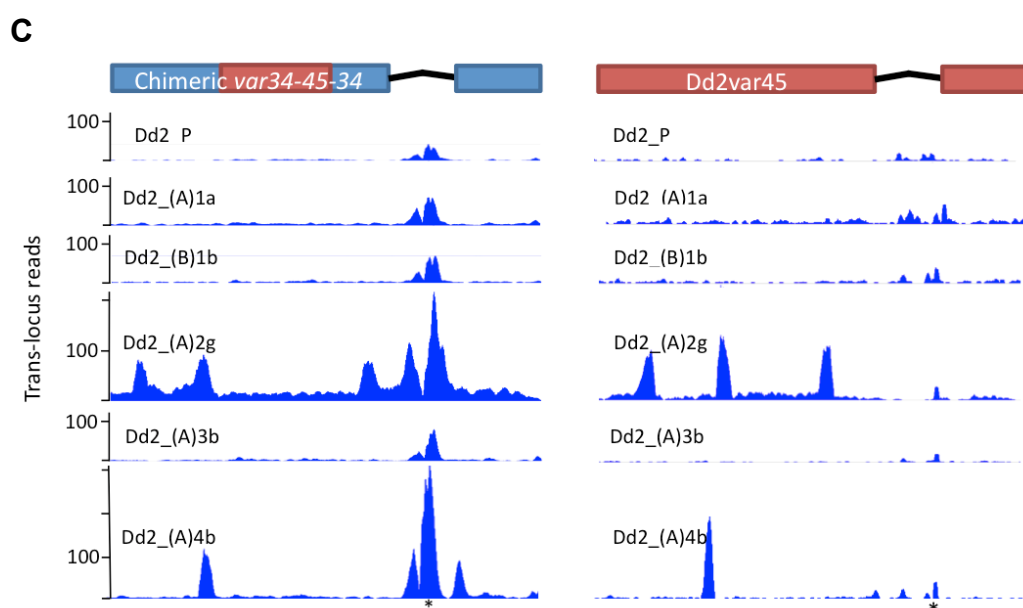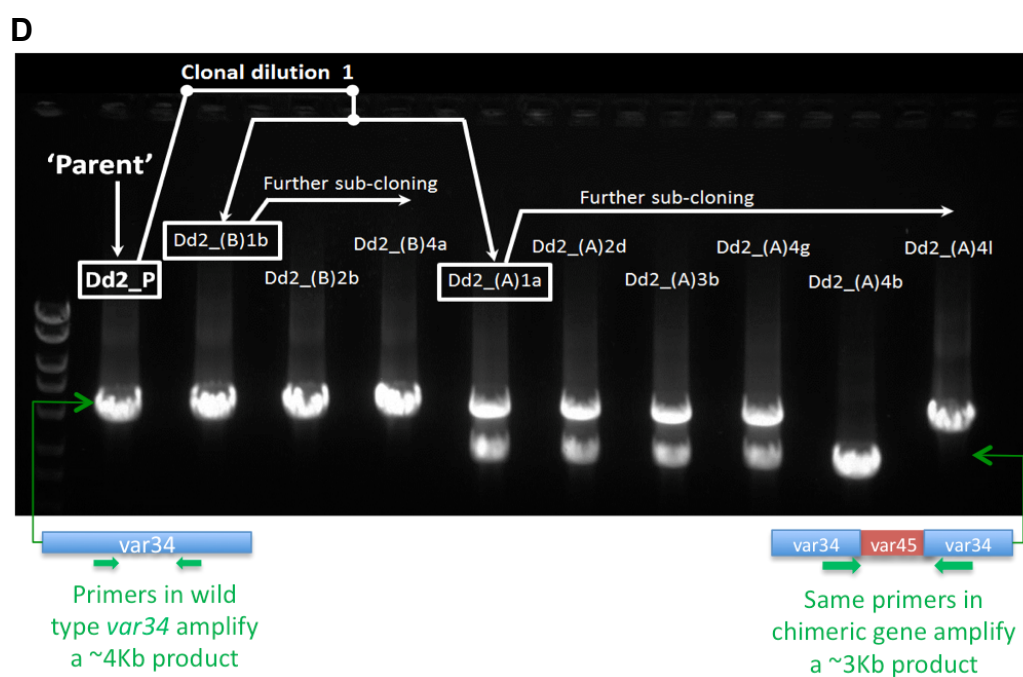

Supplement: S7 Fig — Evolution of a var gene. See also Fig. 2 in the main manuscript for a summary. (A) Coverage plots for Dd2var34 and Dd2var45 from LookSeq. The lower part shows trans-locus pair-end reads only. Two peaks are found in sample Dd2_(A)1a and all its progeny (not shown here). The coordinates of these trans-locus peaks correspond exactly to the rise in coverage for Dd2var34 and to the fall in coverage in Dd2var45. (B) Model describing this chromosomal rearrangement: a new chimeric var gene has been created in sample Dd2_(A)1a, the sequence being an hybrid between Dd2var34 and Dd2var45. (C) Trans-locus pair-end reads mapped to a reference genome where the Dd2var34 sequence has been replaced by the chimera Dd2var34-45-34. Note that peaks in Dd2_(A)1a have now disappear. In two of its progeny, Dd2_(A)2g and Dd2_(A)4b, 3 and 1 new translocation peaks have appeared, respectively. (D) PCR with primers overlapping the translocated sequence. In Dd2var34, the primers give a 3974 bp product, which is seen in all clones. In the chimeric var, the primers give a smaller product of 2756 bp, seen only in sample Dd2_(A)1a and its progeny. As predicted, clone Dd2(A)4b has lost Dd2var34 while clone DD2_(A)4 l has lost the chimera Dd2var34-45-34. * Unspecific single read peaks found in all samples. (PDF) [file pgen.1004812.s007.pdf]

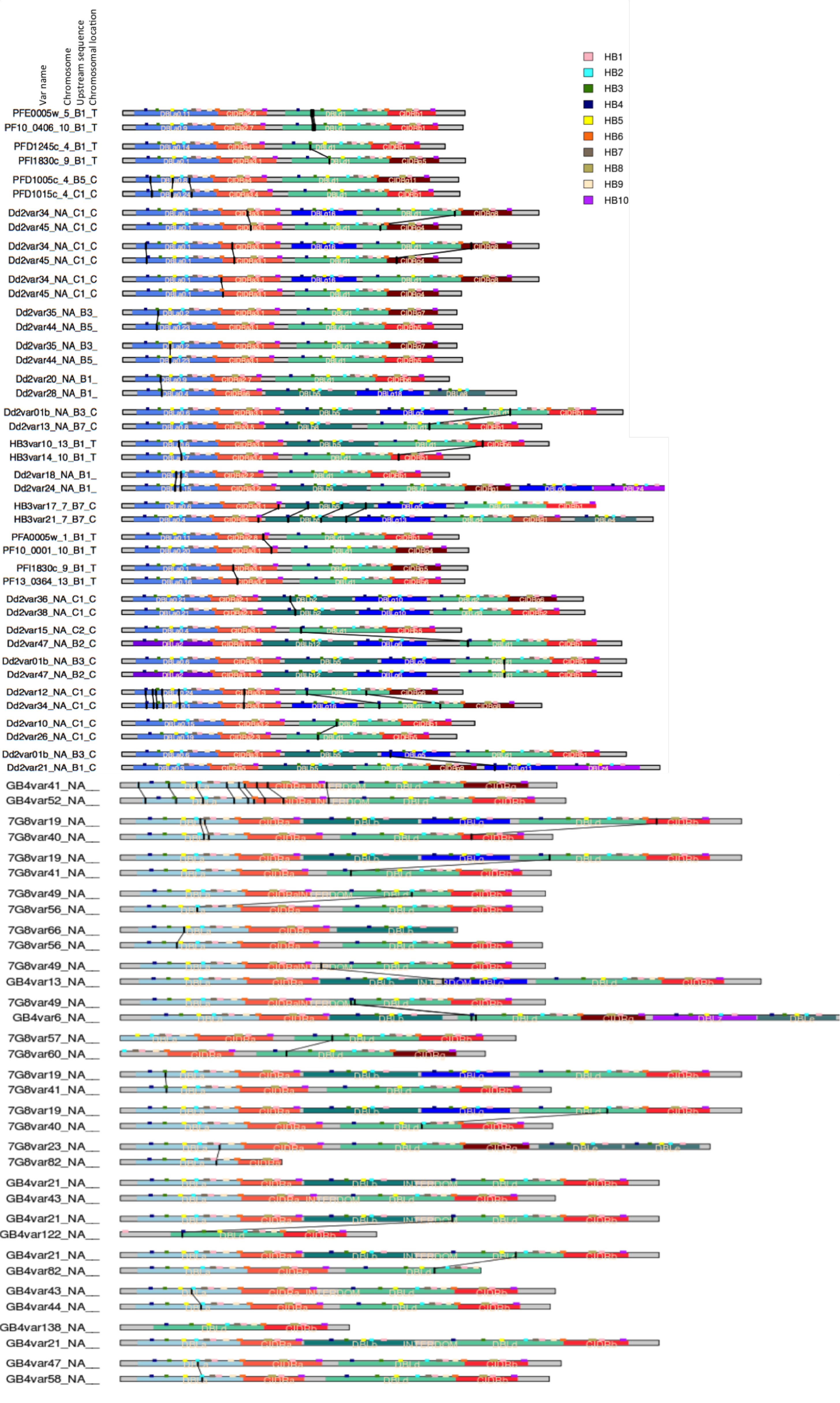

Supplement: S8 Fig — All var exon 1 translocations identified in this study. Recombination breakpoints are marked by a vertical back line, and linked with the recombining var. The data for this figure can be found in S4 Table. For simplicity, only homology blocks 1 to 10 are represented here, with small colour rectangles above each domain. In two instances, the coverage of trans-locus reads did not allow the identification of the recombination breakpoint coordinates (as it is normally done in S4 Fig. for example), hence the lack of black lines between GB4var21-GB4var43, and GB4var128-GB4var21. NA = unknown chromosomal location. (TIF) [file pgen.1004812.s008.tif]

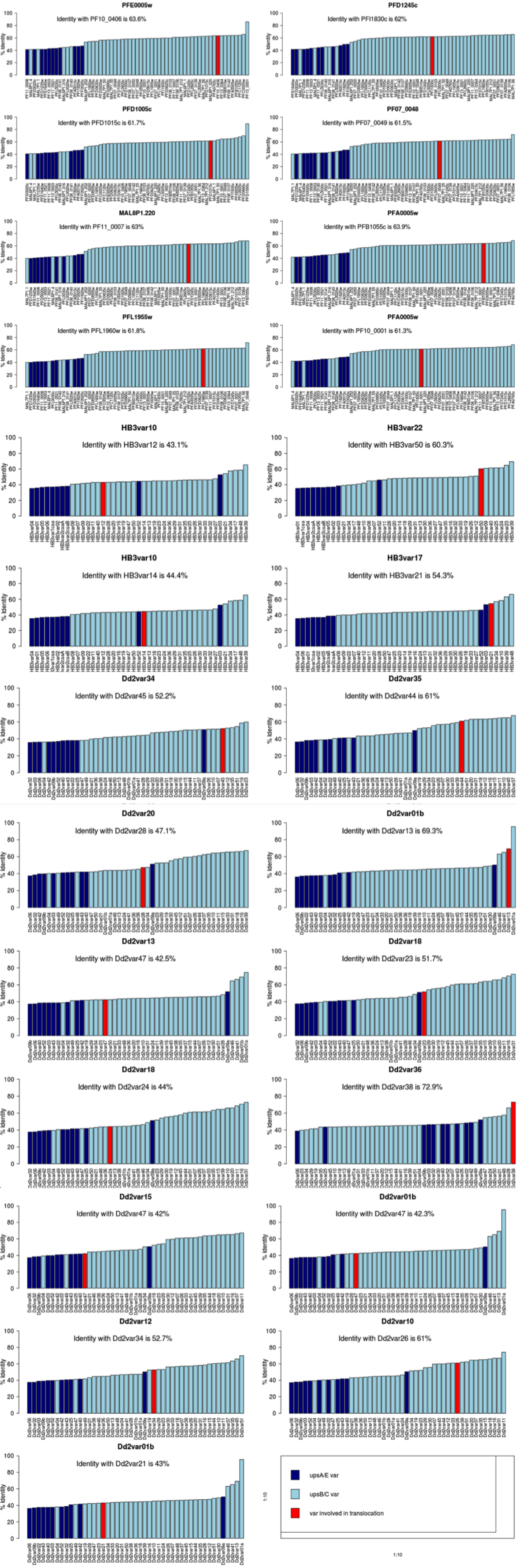

Supplement: S9 Fig — Group B/C var genes do not necessarily recombine with var genes of highest similarity. Var genes were pairwise aligned within each strain. Percentage of nucleotide identity of the recombining var is shown in red. Group A and E var genes, where no recombination had been observed in our dataset, are shown in dark blue. Nucleotide identities between group B/C var genes ranged from 53 to 99% (average = 66.8%). The two recombining exon 1 var genes had an average of 63% Identity (range 42 to 73%). Data from the 7G8 and GB4 strains is not represented here as their var genes are not fully assembled. (TIF) [file pgen.1004812.s009.tif]

A

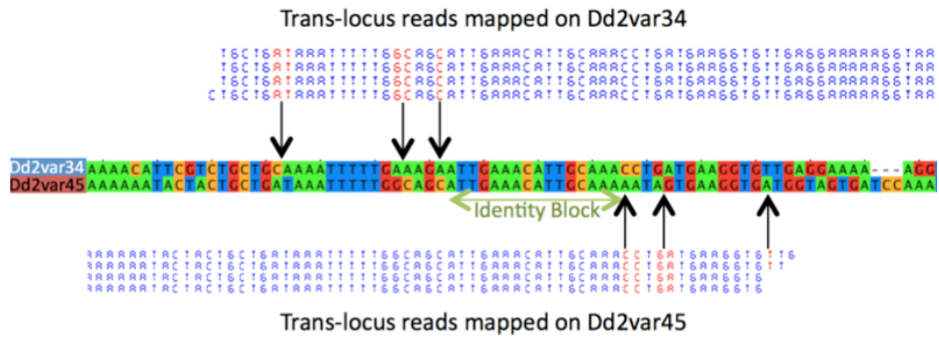

B

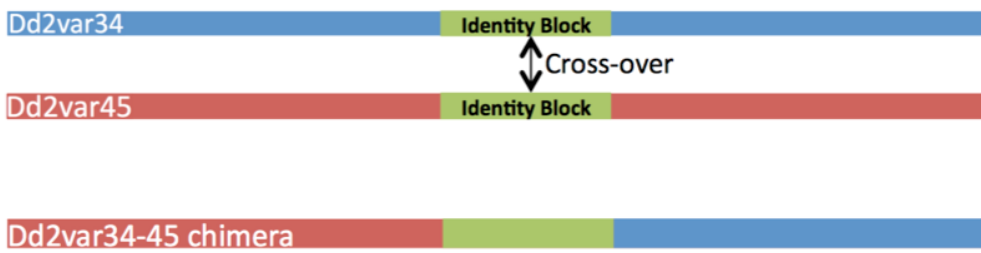

Supplement: S10 Fig — Defining identity blocks. Var B/C genes recombine (A) ClustalW alignment of Dd2var34 and Dd2var45 at a crossover. Example sequence reads mapped to Dd2var34 and Dd2var45 are shown above and below the alignment respectively. Sequence reads are coloured blue when they match the reference and red when there is a mismatch. All mismatches near the recombination breakpoint correspond to the sequence from the other var that has been translocated. The sequence between the mismatches is where the crossing-over event must have occurred, and is by definition identical between both var genes. We term these sequences ‘identity blocks’. (B) Cartoon demonstrating formation of ‘Dd2var34/45’ chimera, with recombination breakpoint occurring within the identity block. (PDF) [file pgen.1004812.s010.pdf]

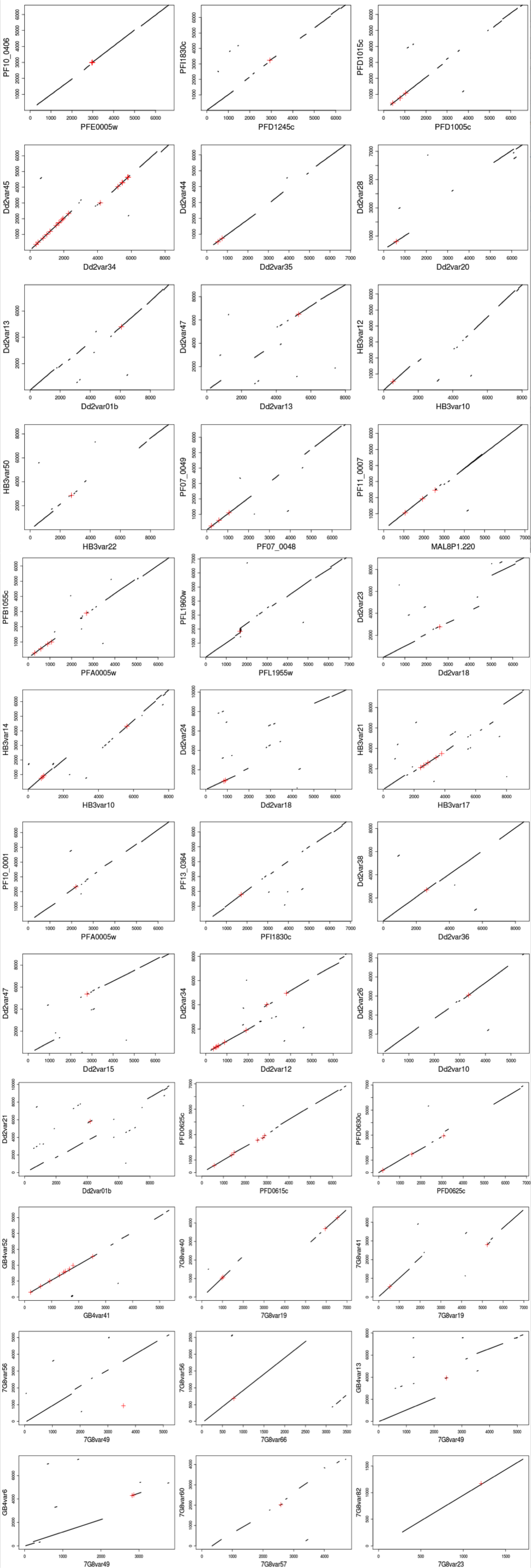

Supplement: S11 Fig — Recombination in var genes occurs where sequences are similar. Dot plot matrix with each pair of recombining var genes indicate the length of each BLAST hit (black line). Location of recombination breakpoints are indicated by red crosses. BLAST was run using default parameters. (TIF) [file pgen.1004812.s011.tif]

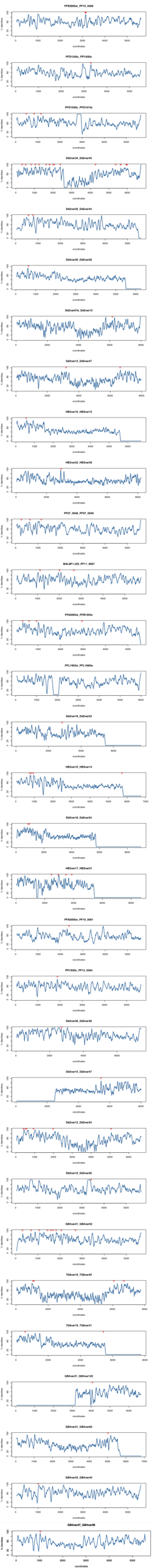

Supplement: S12 Fig — Var gene exon 1 recombine in short regions of higher homology. Each plot is a ClustalW2 DNA alignment (default parameters) of a pair of recombining var genes. The Y-axis shows the percentage of identities (overlapping 50 bp windows) between the two sequences. The red triangles indicate where the recombination breakpoint was found. Plots without any triangle mean that the precise breakpoint coordinate could not be identified. (TIF) [file pgen.1004812.s012.tif]

A

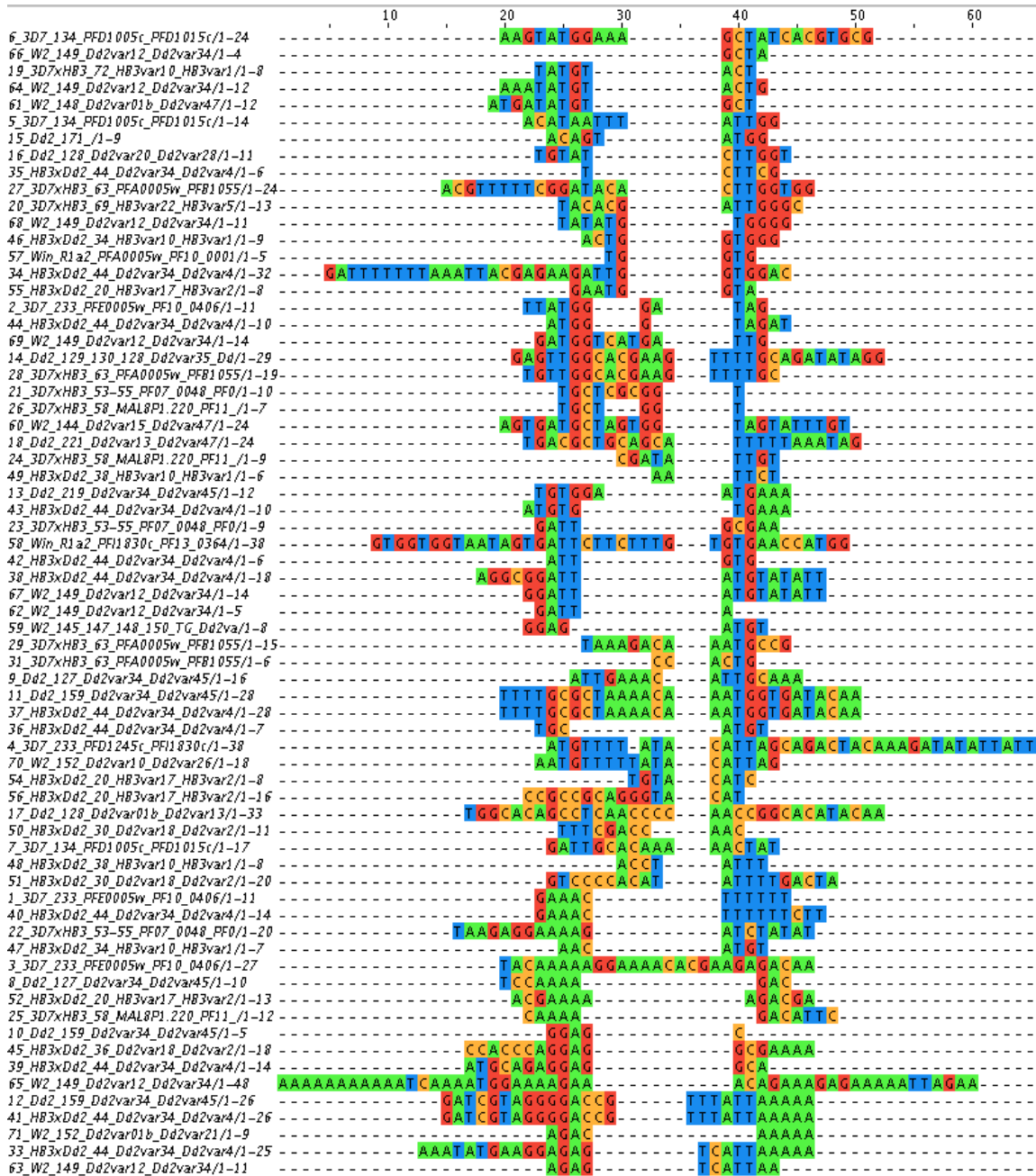

B

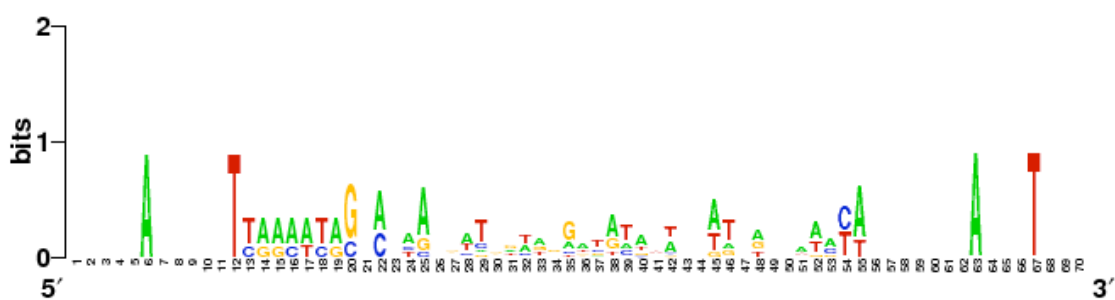

C

## Motif Overview

---

### [Motif 1](#)

- 4.1e+011
- 57 sites

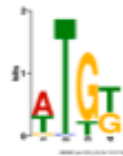

### [Motif 2](#)

- 3.3e+055
- 57 sites

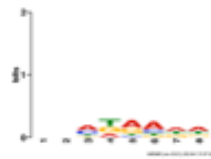

### [Motif 3](#)

- 3.7e+062
- 57 sites

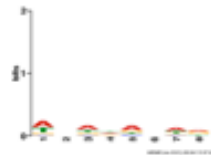

Supplement: S15 Fig — No conserved motifs within Identity Blocks. (A) ClustalW alignment of 71 Identity Blocks. (B) LOGO representation of the most conserved nucleotides. Note that the ‘A’ and ‘T’ at positions 6, 12, 62 and 67 represent only two identity block sequences, i.e. they are not particularly conserved. (C) Top 3 most common motifs identified by MEME, using Identity Blocks ≥8 bp. (PDF) [file pgen.1004812.s015.pdf]
